# Supplementary material for: Artificial intelligence guided discovery of a barrier-protective therapy in inflammatory bowel disease
Source: Nat Commun. 2021 Jul 12;12:4246. doi: 10.1038/s41467-021-24470-5 (PMC8275683; doi:10.1038/s41467-021-24470-5)
Supplement: Supplementary file 1 — Supplementary Information [file 41467_2021_24470_MOESM1_ESM.pdf]

Supplementary Materials for

Artificial Intelligence guided Discovery of a Barrier-Protective Therapy in Inflammatory Bowel Disease

Authors: Debashis Sahoo[1-3†]*, Lee Swanson[4†], Ibrahim M. Sayed[5†], Gajanan D. Katkar[4], Stella-Rita Ibeawuchi[5], Yash Mittal[6], Rama F. Pranadinata[4], Courtney Tindle[4], Mackenzie Fuller[4], Dominik Stec[4], John T. Chang[6], William J. Sandborn[6], Soumita Das[5]* and Pradipta Ghosh[3, 4, 6, 7]*

Affiliations:
[1]Department of Pediatrics, University of California San Diego.
[2]Department of Computer Science and Engineering, Jacob's School of Engineering, University of California San Diego.
[3]Rebecca and John Moore Comprehensive Cancer Center, University of California San Diego.
[4]Department of Cellular and Molecular Medicine, University of California San Diego.
[5]Department of Pathology, University of California San Diego.
[6]Department of Medicine, University of California San Diego.
[7]Veterans Affairs Medical Center, La Jolla, CA.

*Correspondence to:
Debashis Sahoo, Ph.D.; Associate Professor, Department of Pediatrics, University of California San Diego; 9500 Gilman Drive, MC 0703, BRF II - 2119; La Jolla, CA 92093-0703.
Phone: 858-246-1803: **Fax:** 858-246-0019: **Email:** dsahoo@ucsd.edu

Soumita Das, Ph.D.; Associate Professor, Department of Pathology, University of California, San Diego; 9500 Gilman Drive, (MC 0644), George E. Palade Bldg, Rm 256, San Diego, CA, 92093-0644.
Phone: 858-246-2062 (office): **Email:** sodas@ucsd.edu

Pradipta Ghosh, M.D.; Professor, Departments of Medicine and Cell and Molecular Medicine, University of California San Diego; 9500 Gilman Drive (MC 0651), George E. Palade Bldg, Rm 232; La Jolla, CA 92093.
Phone: 858-822-7633: **Fax:** 858-822-7636: **Email:** prghosh@ucsd.edu

† Equal contribution

This PDF file includes:

- **Materials and Methods**
- Supplementary Text- - **Not Applicable**
- **Supplementary Figures. S1 to S19**
- **Supplementary Tables 1 to 3** (separately uploaded)
- **Supplementary Data 1 to 4** (separately uploaded)
- Captions for Movies – **Not Applicable**
- Captions for Audio - **Not Applicable**
- Captions for External Data - **Not Applicable**

Materials and Methods

Computational Approaches

Inflammatory bowel disease (IBD) datasets used for network analysis

A large RNA Seq dataset (Peters 2017, colon tissue; GSE83687; n = 134, 60 control, 32 Ulcerative Colitis, and 42 Crohn's disease) (*1*), another large microarray dataset (Arijs 2018, colon tissue, GSE73661, n = 178, 12 Control, 120 biopsies were from UC VDZ group 41 patients, 46 biopsies from UC IFX group 23 patient) (*2*), a test dataset (Wu 2007, colon tissue, GSE6731, n = 36, 4 normal, 5 UC, 7 CD, 20 other uninvolved and inflamed samples), and other validation dataset (**Fig S2A**, **Supplementary Data 1**) were downloaded from National Center for Biotechnology Information (NCBI) Gene Expression Omnibus website (GEO) (*3-5*). All gene expression datasets (**Supplementary Data 1**) were processed separately using the Hegemon data analysis framework (*6-8*). We did not combine datasets that belong to two different platforms. See **Supplementary Data 1** for the degree of heterogeneity among samples in the dataset.

Test cohort selection

Three test cohorts are selected to build the network and perform machine learning: GSE83687, GSE73661, and GSE6731. GSE83687 is a large RNA Seq dataset and it is the only dataset where a 'full thickness' colon wall analysis was done in the adult IBD cohort. Therefore, this dataset represents the diversity of tissue microenvironments and tissue cell types that are involved in transmural processes (not just mucosa). RNASeq provided high-quality measurements of mRNA extracted from the tissue sample. Since the number of samples in this cohort is 134, which is on the lower side for comprehensive Boolean analysis, we filtered the relationships with another cohort, GSE73661 (n = 178) which is a large microarray dataset on colon mucosa. Machine learning is performed on the third dataset, GSE6731 (n = 36) to cover the entire spectrum of gene expression dataset from old microarrays to modern RNA Seq data.

Boolean Analysis

Boolean logic is a simple mathematic relationship of two values, i.e., high/low, 1/0, or positive/negative. The Boolean analysis of gene expression data requires first the conversion of expression levels into two possible values. The *StepMiner* algorithm is reused to perform Boolean analysis of gene expression data (*9*). The *Boolean analysis* is a statistical approach which creates binary logical inferences that explain the relationships between phenomena. Boolean analysis is performed to determine the relationship between the expression levels of pairs of genes. The *StepMiner* algorithm is applied to gene expression levels to convert them into Boolean values (high

and low). In this algorithm, first the expression values are sorted from low to high and a rising step function is fitted to the series to identify the threshold. Middle of the step is used as the *StepMiner* threshold. This threshold is used to convert gene expression values into Boolean values. A noise margin of 2-fold change is applied around the threshold to determine intermediate values, and these values are ignored during Boolean analysis. In a scatter plot, there are four possible quadrants based on Boolean values: (low, low), (low, high), (high, low), (high, high).

Invariant Boolean implication relationships

A Boolean implication relationship is observed if any one of the four possible quadrants or two diagonally opposite quadrants are sparsely populated. Based on this rule, there are six different kinds of Boolean implication relationships. Two of them are symmetric: equivalent (corresponding to the highly positively correlated genes), opposite (corresponding to the highly negatively correlated genes). Four of the Boolean relationships are asymmetric and each corresponds to one sparse quadrant: (low => low), (high => low), (low => high), (high => high). BooleanNet statistics (Equations listed below) is used to assess the sparsity of a quadrant and the significance of the Boolean implication relationships (*9, 10*). Given a pair of genes A and B, four quadrants are identified by using the StepMiner thresholds on A and B by ignoring the Intermediate values defined by the noise margin of 2 fold change (+/- 0.5 around StepMiner threshold). Number of samples in each quadrant are defined as a_{00}, a_{01}, a_{10}, and a_{11}. Total number of samples where gene expression values for A and B are low is computed using following equations.

$$nA_{low} = (a_{00} + a_{01}), nB_{low} = (a_{00} + a_{10}),$$

Total number of samples considered is computed using following equation.

$$total = a_{00} + a_{01} + a_{10} + a_{11}$$

Expected number of samples in each quadrant is computed by assuming independence between A and B. For example, expected number of samples in the bottom left quadrant $e_{00} = \hat{n}$ is computed as probability of A low ((a_{00} + a_{01})/total) multiplied by probability of B low ((a_{00} + a_{10})/total) multiplied by total number of samples. Following equation is used to compute the expected number of samples.

$$n = a_{ij}, \hat{n} = (nA_{low}/total * nB_{low}/total) * total$$

To check whether a quadrant is sparse, a statistical test for (e_{00} > a_{00}) or ($\hat{n} > n$) is performed by computing S_{00} and p_{00} using following equations. A quadrant is considered sparse if S_{00} is high ($\hat{n} > n$) and p_{00} is small.

$$S_{ij} = \frac{\hat{n} - n}{\sqrt{\hat{n}}}$$

$$p_{00} = \frac{1}{2}\left(\frac{a_{00}}{(a_{00} + a_{01})} + \frac{a_{00}}{(a_{00} + a_{10})}\right)$$

A threshold of S_{00} > sthr and p_{00} < pthr to check sparse quadrant. A Boolean implication relationship is identified when a sparse quadrant is discovered using following equation.

$$\textbf{\textit{Boolean Implication}} = (S_{ij} > \text{sthr}, p_{ij} < \text{pthr})$$

A relationship is called Boolean equivalent if top-left and bottom-right quadrants are sparse.

$$Equivalent = (S_{01} > sthr,\ P_{01} < pthr, S_{10} > sthr, P_{10} < pthr)$$

Boolean opposite relationships have sparse top-right (a_{11}) and bottom-left (a_{00}) quadrants.

$$Opposite = (S_{00} > sthr,\ P_{00} < pthr, S_{11} > sthr, P_{11} < pthr)$$

Boolean equivalent and opposite are symmetric relationship because the relationship from A to B is same as from B to A. Asymmetric relationship forms when there is only one quadrant sparse (A low => B low: top-left; A low => B high: bottom-left; A high=> B high: bottom-right; A high => B low: top-right). These relationships are asymmetric because the relationship from A to B is different from B to A. For example, A low => B low and B low => A low are two different relationships.

A low => B high is discovered if bottom-left (a_{00}) quadrant is sparse and this relationship satisfies following conditions.

$$A\ low \Rightarrow B\ high = (S_{00} > sthr,\ P_{00} < pthr)$$

Similarly, A low => B low is identified if top-left (a_{01}) quadrant is sparse.

$$A\ low \Rightarrow B\ low = (S_{01} > sthr,\ P_{01} < pthr)$$

A high => B high Boolean implication is established if bottom-right (a_{10}) quadrant is sparse as described below.

$$A\ high \Rightarrow B\ high = (S_{10} > sthr,\ P_{10} < pthr)$$

Boolean implication A high => B low is found if top-right (a_{11}) quadrant is sparse using following equation.

$$A\ high \Rightarrow B\ low = (S_{11} > sthr,\ P_{11} < pthr)$$

For each quadrant, a statistic S_{ij} and an error rate p_{ij} is computed. $S_{ij} > 2.5$ and $p_{ij} < 0.1$ are the thresholds used on the BooleanNet statistics to identify Boolean implication relationships (BIRs). False discovery rate is computed by randomly shuffling each gene and computing the ratio of the number of Boolean implication relationship discovered in the randomized dataset and original dataset. For IBD dataset the false discovery rate was less than 0.001.

Boolean Implication analysis looks for invariant relationship across all the different types of samples regardless of the conditions and treatment protocols. Therefore, it does not distinguish the sample types when discovering Boolean implication relationships. We assume that there are fundamental invariant Boolean implication formula that are satisfied by every sample regardless of their type (in this context it is limited to healthy and IBD colonic biopsies including both UC and CD). This means normal, UC and CD samples share the same fundamental relationships.

Preparation and validation of IBD datasets for analysis

Both Peters-2017 and Arijs-2018 dataset were indepndently prepared for Boolean analysis by filtering genes that have reasonable dynamic range of expression values by analyzing the fraction of high and low values identified by the StepMiner algorithm (*11*). Any probeset or genes that contain less than 5% of high or low values are dropped from the analysis. To check if pairwise Boolean implication relationships are consistent between two datasets, every gene in Peters-2017 dataset is mapped to the best probeset (identified by the biggest dynamic range) in the Arijs-2018 dataset, and genes/probesets that do not match are dropped from the analysis. Since RNA-Seq expression values have slightly different characteristics than microarray expression values, the consistency of Boolean implication relationship was determined by using BooleanNet statistics in both datasets and a Pearson's correlation coefficient in the Arijs-2018 dataset. A Pearson's correlation coefficient > 0.5 was considered compatible with Equivalent, High => High, and Low => Low Boolean implication relationships. Similarly, a Pearson's correlation coefficient < -0.25 was considered compatible with Opposite, High => Low, and Low => High Boolean implication relationships.

Construction of a Network of Boolean Implications

A Boolean implication network (BIN) is created by identifying all significant pairwise Boolean implication relationships (BIRs) that are consistent in both Peters-2017 GSE83687 and Arijs-2018 GSE73661 datasets independently (**Fig S1A**) (*1, 12*). The Boolean implication network contains the six possible Boolean relationships between genes in the form of a directed graph with nodes as genes and edges as the Boolean relationship between the genes. The nodes in the BIN are genes and the edges correspond to BIRs. Equivalent and Opposite relationships are denoted by undirected edges and the other four types (low => low; high => low; low => high; high => high) of BIRs are denoted by having a directed edge between them. The network of equivalences seems to follow a scale-free trend; however, other asymmetric relations in the network do not follow scale-free properties. BIR is strong and robust when the sample sizes are usually more than 200 (from our experience of using Boolean Implication for more than 10 years). All our previous papers use thousands of diverse samples to establish Boolean implication relationships. Boolean Implication analysis is carried out for the first time in such low number of samples such as the selected IBD GSE83687 dataset (n = 134). We have demonstrated that we have a reasonable False Discovery Rate (< 0.001) when S > 2.5 and p < 0.1 are used. The IBD dataset was prepared for Boolean analysis by filtering genes that had a reasonable dynamic range of expression values. When the dynamic range of expression values was small, it was difficult to distinguish if the values were all low or all high or there were some high and some low values. Thus, it was determined to be best to ignore them during Boolean analysis. The filtering step was performed by analyzing the fraction of high and low values identified by

the StepMiner algorithm (*11*). Any probe set or genes which contained less than 5% of high or low values were dropped from the analysis.

Generation of Clustered Boolean Implication network

Clustering was performed in the Boolean implication network to dramatically reduce the complexity of the network (**Fig S1B**). A clustered Boolean implication network (CBIN) was created by clustering nodes in the original BIN by following the equivalent BIRs. One approach is to build connected components in an undirected graph of Boolean equivalences. However, because of noise, the connected components become internally inconsistent e.g., two genes opposite to each other become part of the same connected component. In addition, the size of clusters became unusually big with almost everything in one cluster. To avoid such a situation, we need to break the component by removing the weak links. To identify the weakest links, we first computed a minimum spanning tree for the graph and computed the Jaccard similarity coefficient for every edge in this tree. Ideally if two members are part of the same cluster, they should share as many connections as possible. If they share less than half of their total individual connections (Jaccard similarity coefficient less than 0.5) the edges are dropped from further analysis. Thus, many weak equivalences were dropped using the above algorithm leaving the clusters internally consistent. We removed all edges that have Jaccard similarity coefficient less than 0.5 and built the connected components with the rest. The connected components were used to cluster the BIN which is converted to the nodes of the CBIN. The distribution of cluster sizes was plotted in a log-log scale to observe the characteristic of the Boolean network (**Fig S1C**). The cluster sizes were distributed along a straight line in a log-log plot suggesting scale-free properties. The choice of the threshold on the Jaccard similarity coefficient play an important role in determining the size and the number of clusters as well as whether they are internally consistent. We found that a threshold of 0.5 gave us a reasonable number of clusters and followed a scale-free distribution in the cluster sizes. A bigger threshold such as 0.7 to 0.9 will be very aggressive and reduce the cluster sizes (almost all edges will be dropped). A smaller number such as 0.4 will tend to make a bigger cluster with an unusual distribution of cluster sizes. A new graph was built that connected the individual clusters to each other using Boolean relationships. The link between two clusters (A, B) was established by using the top representative node from A that was connected to most of the members of A and sampling 6 nodes from cluster B and identifying the overwhelming majority of BIRs between the nodes from each cluster.

A CBIN was created using the selected Peters-2017 GSE83687 and Arijs-2018 GSE73661 datasets. Each cluster was associated with healthy or disease samples based on where these gene clusters were highly expressed. The edges between the clusters represented the Boolean relationships that are color-coded as follows: orange for low => high, dark blue for low => low, green for high => high, red for high => low, light blue for the equivalent and black for the opposite.

Charting Boolean paths

Boolean paths have been explored before to predict the underlying time series events in biological processes such as B cell differentiation (*10, 13*) and early differentiation events in cancer stem cell (*6-8, 14*). This algorithm is called MiDReG (Mining Developmentally Regulated Genes) that uses two seed genes to identify intermediate genes in a biological process. MiDReG infer intermediate states using a sequence of asymmetric BIRs. Here, using MiDReg algorithm/concept to traverse the Boolean Implication network that identifies paths of clusters where the start and end clusters in the clustered Boolean implication network mark the end points of a possible set of events from healthy to disease. The asymmetric BIRs provide a unique dimension to the network that is fundamentally different from any other gene expression networks in the literature. Traversing a set of nodes in a directed graph of the Boolean network constitutes a Boolean path that can be interpreted as follows. A simple Boolean path involves two nodes and the directed edge between them. This simple Boolean path can be interpreted as shown in the supplementary figure (**Fig S1D**). For the nodes X and Y with X low => Y low only quadrant #1 is sparse; the other quadrants #0, #2, and #3 are filled with samples (**Fig S1D**). Assuming monotonicity in X and Y, the quadrants can be ordered in two possible ways: 0-2-3 and 3-2-0. The path corresponds to 0-2-3 begins with X low and Y low. This is interpreted as X turns on first and then Y turns on along a hypothetical biological path defined by the sample order. Similarly, Y turns off first and then X turns off in the path 3-2-0. A complex path in the Boolean network involves more than one Boolean implication relationship (**Fig S1E**). Three Boolean implication relationships can be used to group samples into five bins and the bins can be ordered in two possible ways (**Fig S1E**, forward, reverse). Another example of a path is illustrated in the supplementary figure (**Fig S1F**). These paths might represent underlying time-series events as was represented in B cell differentiation using MiDReG algorithm (*10*). Once the path to be queried is identified, we then ask which end has more healthy or more diseased samples. Based on the orientation of the path (i.e., healthy vs disease end), and the concept that there are time series of events in any biological data, it is hypothesized that our algorithm might identify some of the underlying characteristics of the time series events of disease progression.

Discovery of Paths in Clustered Boolean Implication network

Discovery of paths start with a node that represents the biggest cluster in the CBIN. Since a path of high=>high, high=>low, and low=>low can be used to order samples as shown in supplementary figure S1E, we try to identify paths of this type that intersects the big clusters in the network. We developed a simple, intuitive algorithm that traverses the nodes of the CBIN starting with the biggest cluster and greedily chooses next big cluster connected to the nodes visited in sequence. The emphasis on cluster sizes comes from the fundamental assumption that size determines importance and relevance. Therefore, we start from a big cluster (A1) and identify other clusters that form a chain of low => low. Further, we identify other clusters that are either opposite to A1 or they have

high=>low relationship with A1, and the biggest cluster (A2) among these clusters was chosen. In addition, a chain of low=> low relationship from A2 is identified. In each subsequent step, again the biggest cluster among the different choices was greedily chosen. Finally equivalence relationship from each cluster is used to gather more genes in each cluster and the whole path is clustered based on equivalence relationships. Depth-first traversal (DFS) was used to follow the path of low => low where bigger clusters are visited first. The search was performed until a cluster was reached for which there is no low => low relationships. For example, starting with cluster S, the search will return S low => A1 low, A1 low => A2 low, and A2 low => A3 low if A3 doesn't have any low => low relationships. Similarly, a new starting point is considered S2 such that S2 is the biggest cluster X that have either S high => X low or S Opposite X. From cluster S2 another DFS was performed to retrieve the longest possible path of low => low. The search may return S2 low => B1 low, B1 low => B2 low if B2 doesn't have any low => low relationships. In summary, the most prominent Boolean path was discovered by starting with the largest cluster and then exploring edges that connected to the next largest cluster in a greedy manner. This process was repeated to explore paths that connects the big clusters in the network.

Scoring Boolean path for sample order

A score was computed for a specified Boolean path that can be used to order the sample which was consistent with the logical order. To compute the final score, first the genes present in each cluster were normalized and averaged. Gene expression values were normalized according to a modified Z-score approach centered around StepMiner threshold (formula = (expr - SThr)/3*stddev). A weighted linear combination of the averages from the clusters of a Boolean path was used to create a score for each sample. The weights along the path either monotonically increased or decreased to make the sample order consistent with the logical order based on BIR. The samples were ordered based on the final weighted and linearly combined score. The direction of the path was derived from the connection from a healthy cluster to a disease cluster.

Summary of genes in the clusters

Reactome pathway analysis of each cluster along the top continuum paths was performed to identify the enriched pathways (*15*). The pathway description was used to summarize at a high-level what kind of biological processes are enriched in a particular cluster.

Assessing the association of IBD signature genes with AMPK subunits

The association between mRNA expression levels of various AMPK subunits and Claudins was tested in a cohort previously reported (*1*). This cohort included gene expression data from multiple publicly available NCBI-GEO data-series (GSE100833, GSE83550, GSE83687). To investigate the relationship between the mRNA expression

levels of selected genes (i.e. PRKAB1 and CLDN2), we applied the *Hegemon*, "*hierarchical* exploration of *gene expression* microarrays on-line" tool (*6*). The Hegemon software is an upgrade of the *BooleanNet* software (*9*), where individual gene-expression arrays, after having been plotted on a two-axis chart based on the expression levels of any two given genes, can be stratified using the *StepMiner* algorithm (*11*) and compared for statistically significant differences in expression. We stratified the patient population of the NCBI-GEO discovery dataset in different gene-expression subgroups, based on the mRNA expression levels of various AMPK subunits, and compared the expression of IBD-associated genes between groups.

Generation of heat maps using gene clusters identified by Boolean analysis

To generate the IBD, UC and CD heatmaps (**Fig S1G**) first a Boolean path was constructed by following the largest clusters in the Boolean Network (*1, 12*). Genes along this path were selected to generate a heatmap that shows the gene expression values in different samples. To build heatmaps using the datasets from patients treated with either an anti-TNF (Infliximab; GSE16879; **Fig S4**), (*12*) or anti-α4β7 (Vedolizumab; GSE73661; **Fig S5**) (*2*), the gene clusters (C1-2-3) along the major IBD-paths were used. Gene expression values were normalized according to a modified Z-score approach centered around *StepMiner* threshold (formula = (expr - SThr)/3*stddev). The samples were ordered according to the average of the normalized gene expression values in the largest cluster along the Boolean path. The heatmap uses red colors for the high values, white colors for the intermediate values and blue colors for low values. Gene names for few selected genes are highlighted on the left to show their expression patterns.

Identification of Epithelial-Mesenchymal and Inflammation-Fibrosis continuum

Top genes involved with Epithelial-Mesenchymal processes and inflammation-fibrosis processes are chosen from the literature review. Given a list of genes BoNE computes a subgraph of the CBIN graph by identifying clusters that include one or more genes from this list. BoNE then searches for a path in this subgraph as mentioned before with the original CBIN graph. The path identified is used to draw a model of the gene expression timeline (**Fig S1D**). The continuum is identified by computing a score based on the path as described before.

Measurement of classification strength or prediction accuracy

Receiver operating characteristic (ROC) curves were computed by simulating a score based on the ordering of samples that illustrates the diagnostic ability of binary classifier system as its discrimination threshold is varied along with the sample order. The ROC curves were created by plotting the true positive rate (TPR) against the false positive rate (FPR) at various threshold settings. The area under the curve (often referred to as simply the AUC) is equal to the probability that a classifier will rank a randomly chosen IBD samples higher than a randomly

chosen healthy samples. In addition to ROC AUC, other classification metrics such as accuracy ((TP + TN)/N; TP: True Positive; TN: True Negative; N: Total Number), precision (TP/(TP+FP); FP: False Positive), recall (TP/(TP+FN); FN: False Negative) and f1 (2 * (precision * recall)/(precision + recall)) scores were computed. Precision score represents how many selected items are relevant and recall score represents how many relevant items are selected. Fisher exact test is used to examine the significance of the association (contingency) between two different classification systems (one of them can be ground truth as a reference).

AI guided discovery of Boolean paths

A Boolean path is converted to a path score as mentioned above using a linear combination of normalized gene expression values. The strength of classification of healthy and IBD samples using this score is computed by the ROC-AUC measurement. We performed a multivariate regression (**Fig 2E**) to identify the best Boolean path that predicts normal vs IBD samples in the cohort GSE6731 (4 N, 5 UC, 7 CD). We tested how the path score distinguishes healthy and IBD samples as they are annotated in many other independent colon-derived datasets.

Comparison of Boolean with Bayesian and Differential analyses

We used 133 key driver genes (KDG) predicted by the Bayesian analysis from Peters et al. 2017 (*1*). We performed standard t-tests adjusted at 1% FDR using the Benjamini-Hochberg Procedure to identify differentially expressed genes between normal and IBD samples in GSE83687 (171 down- and 162 up-regulated in IBD). A gene signature score is computed for each approach (Boolean, Differential, and Bayesian) by using weighted linear combination of normalized Z-scored expression values as mentioned above. Weights -1 and +1 is used for down and up-regulated genes respectively from the differential analysis to get a combined score. The samples were ordered based on the final weighted and linearly combined score. The sample order is evaluated using the sample annotation (Healthy, IBD) by ROC-AUC analysis.

Test and Validation IBD Datasets

Two datasets (GSE83687, GSE73661) were used to build a Boolean implication network, and GSE6731 is used to train Boolean models that distinguish healthy and IBD samples. A Boolean path is selected after machine learning to construct a Boolean model. In this model a path score is computed as mentioned in section "*Scoring Boolean path for sample order*" that is used to order the samples. The sample order is evaluated using the sample annotation (Healthy, IBD) by ROC-AUC analysis. The Boolean model is tested in several human and mouse datasets, each comprised of a heterogeneous collection of samples (as mentioned in **Supplementary Data 1**) to demonstrate reproducibility. The dataset contained both pediatric and adult IBD samples and represented both genders equally well. Since, the Boolean model created was based on colon-derived datasets, testing was mostly

performed in colon-derived samples selected from each dataset whenever possible. There are several mouse models of IBD available such as DSS(*16*), TNBS(*17-19*), IL10-/-(*20, 21*) and adoptive T-cell transfer models (*22*). We have collected publicly available gene expression datasets derived from mouse models of IBD (**Supplementary Data 1**) to test whether human Boolean models perform well in mice. The gene name conversion from human to the mouse is performed using human genome GRCh38.95 ensembl IDs and mapping data exported from ensemble BioMart web-interface.

Experimental Approaches:

Reagents and antibodies

Unless otherwise indicated, all reagents were of analytical grade and obtained from Sigma-Aldrich (St. Louis,MO). Custom-designed oligos were obtained from Valuegene (San Diego, CA). Antibodies against GIV that were used in this work include rabbit serum anti-GIV coiled-coil immunoglobulin G (GIV-ccAb for immunoblotting only) (*23*), and affinity-purified GIV-cc Ab (Cat# ABT80; from EMD Millipore for immunoblotting). Mouse mAbs against anti-phospho-(p; Cell Signaling Technology, Danvers, MA) and total (t; Abcam, Cambridge, UK) AMPK, anti-Claudin-2 (Abcam), and anti-tubulin (Sigma,St. Louis, MO) were purchased from commercial sources. Rabbit polyclonal antibodies against phospho-S245 GIV were generated commercially by 21st Century Biochemicals (Marlborough, MA) and validated previously (*24*). DAPI and anti-mouse Alexa Fluor 488 or 594–coupled goat secondary antibody for immunofluorescence were purchased from Invitrogen (Carlsbad, CA). Goat anti-rabbit and goat anti-mouse Alexa Fluor 680 or IRDye 800 $F(ab')_2$ for immunoblotting were from LI-COR Biosciences (Lincoln, NE).

RNA extraction and quantitative-(q) PCR

Total RNA was isolated using the Quick-RNA MicroPrep Kit (Zymo Research, USA) according to the manufacture's instruction. RNA was converted into cDNA using the qScript™ cDNA SuperMix (Quantabio). Quantitative RT-PCR (qRT-PCR) was carried out using 2x SYBR Green qPCR Master Mix (Biotool™, USA). The cycle threshold (Ct) of target genes was normalized to 18S rRNA gene. The fold change in the mRNA expression was determined using the $2^{-\Delta\Delta C_t}$ method. Primers used in qPCR reactions were designed using NCBI Primer Blast software and Roche Universal Probe Library Assay Design software (see Table below).

qPCR primer sequences

Target	Forward primer (5'→3')	Reverse primer (3'→5')
**Human PRKAB1**	tgtcggtttatcttcgcgcc	ctctcgcaatcgcgctttac
**Human CLDN2**	acctgctaccgccactctgt	ctccctggcctgcattatctc

**Human 18S rRNA**	gtaacccgttgaaccccatt	ccatccaatcggtagtagcg

Immunoblotting

For immunoblotting, protein samples were separated by SDS-PAGE and transferred to PVDF membranes (Millipore, Burlington, MA). Membranes were blocked with PBST supplemented with 5% nonfat milk (or with 5% BSA when probing for phosphorylated proteins) before incubation with primary antibodies. Infrared imaging with two-color detection and band densitometry quantifications were performed using the Odyssey imaging system (Li-Cor, Lincoln, NE). All Odyssey images were processed using ImageJ software (NIH, Bethesda, MD.) and assembled into figure panels using Photoshop and Illustrator software suits (Adobe Inc., San Jose, CA.).

Human subjects

Colonic biopsies used either for IHC studies or as a source of stem cells for organoid culture were obtained from IBD patients undergoing colonoscopies a part of their routine care and follow-up at UC San Diego's Inflammatory Bowel Disease (IBD) Center. Patients were recruited and consented using a study proposal approved by the Institutional Review Board of the University of California, San Diego. Isolation and biobanking of organoids from these gastro-intestinal specimens were carried out using an approved human research protocol (IRB# 190105: PI Ghosh and Das) that covers human subject research at the UC San Diego HUMANOID Center of Research Excellence (CoRE). The clinical phenotype and information were curated based on histopathology reports from Clinical Pathology and Chart check, followed by consultation with a specialist at UC San Diego's IBD Center.

For immunohistochemical analysis of human tissue specimens, archived formaldehyde-fixed paraffin-embedded (FFPE) human colonic biopsies from healthy controls, or patients with adenomas and/or carcinomas were obtained from the Gastroenterology Division, VA San Diego Healthcare System, following the protocol approved by the Human Research Protection Program (HRPP) Institutional Review Board (Project ID# 1132632). For the purpose of generating healthy adult enteroids, a fresh biopsy was prospectively collected using small forceps from healthy subjects undergoing routine colonoscopy for colon cancer screening at the VA San Diego Healthcare System. For all the deidentified human subjects the information including age, ethnicity, gender, previous history of disease and medication were collected from the chart following the security and privacy rules outlined in the HIPAA (Health Insurance Portability and Accountability Act of 1996) legislation. Written informed consent was obtained from all participants. The study design and the use of human study participants was conducted in accordance to the criteria set by the Declaration of Helsinki.

Murine models

Intestinal crypts were isolated either from the proximal and the mid-colon of WT C57BL/6 or AMPK KO mice; generated from gender- and age-matched littermates of age 5-7 weeks. For DSS-colitis experiments, 7-8-wk old C57Bl/6 mice were obtained from Jackson Laboratories (Bay Harbor, ME). Animals were bred, housed (light and dark cycle of 12 h each, humidity 30-70% and room temperature controlled between 68-75 °F), and euthanized according to University of California San Diego Institutional Animal Care and Use Committee (IACUC) policies and guidelines.

Isolation of organoids from murine and human colons

Colonic specimens of around 1-inch segment in the case of mice or superficial biopsies in the case of human subjects were collected using cold forceps. The specimens were washed in ice-cold PBS to remove fecal contamination, fat and blood vessels. When acquisition of samples and isolation of stem cells were performed in different facilities, specimens were transported from the site of sample acquisition to the laboratory in media containing DMEM/F12 with HEPES and L-glutamine, 10% FBS and 10 µM Y27632 (ROCK inhibitor). Crypts were isolated by digesting with collagenase type I [2 mg/ml; Invitrogen, Carlsbad, CA] solution containing Gentamicin (50 µg/ml, Life Technologies, Carlsbad, CA) at 37° C by monitoring the digestion of epithelial units up to 80%. The tissue fragments were added to media (DMEM/F12 with HEPES, 10% FBS) to inactivate the collagenase and filtered with a 70 µM cell strainer as outlined before (*25-27*). Filtered tissue fragments were centrifuged down at 100 *g* for 5 min and the media was aspirated. The epithelial units were suspended in matrigel BD basement membrane matrix (Cat# 356235, Corning Costar, Corning, NY). The cell-matrigel suspension (15 µl) was placed at the center of the 24-well plate on ice and placed for 10 m in the incubator upside-down for polymerization. Subsequently, 500 µl of 50% conditioned media (CM) was added. CM was prepared from L-WRN cells (ATCC® CRL-3276™, from the laboratory of Thaddeus S. Stappenbeck (*27*)) with Wnt3a, R-spondin and Noggin. Y27632 (ROCK inhibitor, 10 µM) and SB431542 (an inhibitor for TGF-β type I receptor, 10 µM) were added to the media. For human colon samples, the 50% conditioned media was supplemented with Nicotinamide (10 µM, Sigma-Aldrich, St. Louis, MO), N-acetyl cysteine (1 mM, Sigma-Aldrich), and SB202190 (10 µM, Sigma-Aldrich). The medium was changed every 2-3 d and the enteroids were expanded and frozen in liquid nitrogen. One important caveat to mention is that after extended passage (> 10) of IBD patient-derived enteroids they begin to revert their phenotype to a 'healthy' state (i.e. less disruption of TJs, and higher TEER values). This is likely due to the stress-reducing culture conditions required to propagate the enteroids and therefore it is imperative to use low passage number enteroids when assessing IBD-associated barrier defects.

Preparation of enteroid-derived monolayers (EDMs)

For both murine and human enteroids, polarized EDMs were prepared using a similar protocol outlined below. Single-cell suspensions from typsinized organoids in 5% conditioned media were added to matrigel diluted in cold PBS (1:30) as done before (*28*). 2-4 x 10^5 cells were plated in 24-well trans-well inserts (0.4 µm pore size; Corning Costar, Corning, NY) and differentiated for 2 days in advanced DMEM/F12 media without Wnt3a but with R-Spondin, Noggin, B27 and N2 supplements and 10 µM ROCK inhibitor for the mouse (*26*). For Human EDMs, media and supplements were obtained commercially (Cell Applications Inc. San Diego, CA) and a proprietary cocktail was added to the above media.

Bacteria and bacterial culture

Adherent Invasive *Escherichia coli* strain LF82 (*AIEC-LF82*), isolated initially from the colon of Crohn's disease patients obtained from the lab of Arlette Darfeuille-Michaud (*29*). For bacterial culture, a single colony was inoculated into LB broth and grown for 8 h under aerobic conditions in an orbital shaking incubator at 150 rpm, followed by overnight culture under oxygen-limiting conditions, but without shaking, to maintain their pathogenicity. Cells were infected with a multiplicity of infection (moi) of 10-30 as done before (*30*).

Immunofluorescence

Mouse and human enteroid-derived monolayers (EDMs) were fixed with cold methanol at -20°C for 20 min, washed once with PBS and equilibrated in blocking buffer (0.1% Triton X-100, 2 mg/ml BSA, in PBS) for 1 h. Samples were then incubated with primary and then secondary antibodies as described previously (*31*). Dilutions of antibodies and reagents were as follows: anti-phospho-Ser245-GIV (pS245-GIV; 1:250); anti-Occludin (1:250); DAPI (1:1000); goat anti-mouse (488 and 594 nm wavelength) Alexa-conjugated antibodies (1:500). Images were acquired using a Leica CTR4000 Confocal Microscope with a 63X objective. Z-stack images were obtained by imaging approximately 4-µm thick sections of cells in all channels. Cross-section and maximal projection images were obtained by an automatic layering of individual slices from each Z-stack. Red-Green-Blue (RGB) graphic profiles were created by analyzing the distribution and intensity of pixels of these colors along a chosen line using ImageJ software. All individual images were processed using Image J software and assembled for presentation using Photoshop and Illustrator software (Adobe).

Quantitative (q)PCR analysis of IBD patient samples

Colonic biopsy specimens were collected either from healthy human subjects enrolled for routine colonoscopy or with IBD subjects enrolled for a colonoscopy procedure at the UCSD IBD center. RNA isolation, cDNA

preparation, and analysis of transcript levels for PRKAB1 and CLDN2 were done as described above. Results are displayed as mean ± S.E.M. and p-values calculated using a student two-tailed t-test.

Immunohistochemistry of patient colon samples

Formalin-fixed, paraffin-embedded (FFPE) tissue sections of 4 μm thickness were cut and placed on glass slides coated with poly-L-lysine, followed by deparaffinization and hydration. Heat-induced epitope retrieval was performed using citrate buffer (pH 6.0) in a pressure cooker. Tissue sections were incubated with 0.3% hydrogen peroxidase for 15 m to block endogenous peroxidase activity, followed by incubation with primary antibodies for overnight in a humidified chamber at 4°C. Antibodies used for immunostaining; anti-pS245 GIV [1:50, anti-rabbit antibody], anti-AMPKβ1 [1:50, anti-rabbit], anti-Claudin-2-1 [1:250, anti-rabbit]. Immunostaining was visualized with a labeled streptavidin-biotin using 3,3′-diaminobenzidine as a chromogen and counterstained with hematoxylin. Samples were quantitatively analyzed and scored based on the presence (positive) or absence (negative) of staining. Data is displayed as the frequency of staining score and a Chi-square test was used to determine significance.

Proteomic analysis of IBD patient samples

The proteomic dataset containing healthy and UC patients was obtained from previously published work (*32*). Samples were analyzed for the expression of AMPK subunits and tight junction proteins (occludin). Results are displayed as mean ± S.E.M. and p-values calculated by 2-way ANOVA using Tukey's multiple comparisons test.

Activation of SPS-pathway in colon enteroids by PRKAB1 agonists

3D enteroids were incubated with various chemical activators of AMPK (metformin [1 mM], A-769662 [100 μM], PF-06409577 [1μM]) for 4 h. Enteroids were separated from matrigel by incubating with Cell Recovery Solution (Corning) for 1 h at 4°C with rotation followed by centrifugation at 200 x g for 5 m at 4°C. Media and dissolved matrigel was aspirated and the remaining cell pellet was boiled in Laemelli's sample buffer for 10 m. Samples were analyzed by Western blot, as described above, using: anti-pS245-GIV (1:500, 21st Century Biochemicals, Marlboro, MA), anti-GIV-coiled-coil(CC) (1:500, EMD Millipore), anti-AMPKα and anti-phospho-AMPKα(Thr172) (1:1000, Cell Signaling Technologies, Danvers, MA), and anti-α tubulin (1:1000, Sigma-Aldrich). Quantification of burst tight-junctions was done by manually counting the number of total and burst tri-cellular junctions in 3 randomly chosen fields in each of three independent experiments. Data are expressed as the frequency of burst tight junctions and a one-way ANOVA analysis was used to determine significance.

Measurement of Trans-Epithelial Electrical Resistance (TEER) in 2D-EDM

EDMs were cultured, as described above, on 24-well transwell inserts (0.4 µm pore size; Corning Costar). EDMs were differentiated for 2 d before treatment with various chemical activators of AMPK (metformin [1 mM], A-769662 [100 µM], PF-06409577 [1 µM]) for 16 h. Cultures were then challenged with insults (LPS [500 ng/ml] or *AIEC-LF-82* [moi=10]) and epithelial permeability was measured using an epithelial voltohmmeter Millicel-ERS resistance meter (Millipore) at 1 h intervals for 8 h. TEER was calculated by subtracting measured values from blank control wells and expressed as ohm x cm^2. TEER values were normalized to t_0 and expressed as percentage change relative to t_0. Results are displayed as mean ± S.E.M. and p-values calculated by 2-way ANOVA using Tukey's multiple comparisons test.

Imaging tight junction (TJ) integrity of 2D-EDM by confocal microscopy

EDMs were plated on 24-well transwell inserts, as described above for TEER experiments. After treatment, infection, and measurement of TEER samples were washed once with PBS, pH 7.4 and fixed in 100% methanol (-20°C for 20 m), washed with PBS, and permeabilized/blocked (0.1% Triton-X 100, 2mg/ml BSA in PBS for 1 h at 22°C). Cells were stained using either anti-pS245 GIV (1:300, 21st Century) or anti-Occludin (1:300, Thermo-Fisher, Waltham, MA) overnight at 4°C in blocking solution. For secondary staining goat anti-mouse-Alexa488 (1:500, Life technologies, Carlsbad, CA), goat anti-rabbit-Alexa594 (1:500, Life technologies) and DAPI (1:1000) were prepared in blocking solution and stained for 1 h at 22°C. For imaging, the transwell membranes were cut out and placed cell-side-up on untreated glass microscope slides. ProlongGold™ (20 µl) mounting media (Life technologies) was placed directly on transwell membranes and coverslips were mounted (15 mm, #1 thickness).

Dextran Sodium Sulfate (DSS) mouse model of colitis

Seven week old female C57BL/6 mice obtained from Jackson Laboratories (Bay Harbor, ME) were given either normal drinking water (control) or 3.5% dextran sodium sulfate (DSS) for 5 d, followed by an additional 4 d recovery period with normal drinking water. Water levels were monitored to determine the volume of water consumed by all groups. Weight was monitored daily. Treatment with AMPK agonists (metformin [50 mg/kg/d], A769662 [6 mg/kg/d], PF-06409577 [10 mg/kg/d]) was administered once per day (d0 through d9) via intrarectal injection (50 µl total volume). All compounds were dissolved in 4% DMSO (vehicle). Post-injection, mice were hung upside-down for 30 sec to ensure injection solution was retained in colon. Mice were sacrificed on the 9th day, and colon length was assessed. Colon samples were collected for assessing the levels of mRNA (by qPCR) or proteins (by immunohistochemistry on FFPE tissues) for target genes/proteins. Disease activity index (DAI)

was calculated using by scoring stool consistency (0-4), rectal bleeding (0-4), and weight loss (0-4) as previously published (*33*). Results are displayed as mean ± S.E.M. and p-values calculated by 2-way ANOVA using Tukey's multiple comparisons test. Immunohistochemical analysis was done as described above. Antibodies used for immunostaining; anti-pS245 GIV (1:50, anti-rabbit antibody generated commercially by 21st Century Biochemicals, and extensively validated previously(*24*)), anti-claudin-2 (1:100, anti-rabbit, Abcam, Cambridge, UK), anti-myeloperoxidase (1:30, anti-rabbit, Abcam). Immunostaining was visualized with a labeled streptavidin-biotin using 3,3′-diaminobenzidine as a chromogen and counterstained with hematoxylin. Samples were quantitatively analyzed and scored based on the intensity of staining using the following scale; 0 to 3, where 0 = no staining, 1 = light brown, 2 = brown, and 3 = dark brown. For Periodic Acid Schiff (PAS) staining FFPE tissue sections were first cut into slides, deparaffinized, and rehydrated before immersion into PAS for 5 m at 22°C. Slides were then washed and immersed in Schiff's reagent for 15 m at 22°C. Slides were counterstained in Hematoxylin solution for 3 min, before dehydration and mounting. Hematoxylin and Eosin (H&E) stained slides were evaluated for the presence of neutrophilic and mononuclear infiltrates, submucosal edema, surface erosions, inflammatory exudates, and the presence of crypt abscesses and scored as done previously (*34*). Scoring was carried out by two independent pathologists.

Statistical analyses

Statistical significance between datasets with three or more experimental groups was determined using one-way (or two-way in the case of DSS weight analysis) analysis of variance (ANOVA) including a Tukey's test for multiple comparisons. Statistical difference between two experimental groups was determined using a two-tailed unpaired t-test or two-tailed Mann-Whitney test (patient sample transcript analysis). For analysis of the frequency of SPS-pathway activation in human patient biopsies, a two-tailed Fisher's exact test was used to calculate significance. For all tests, a p-value of 0.05 was used as the cutoff to determine significance. All experiments were repeated a least three times, and p-values are indicated in each figure. All statistical analysis was performed using GraphPad prism 8.

Supplementary Text

Not applicable.

Supplementary Figures and Legends

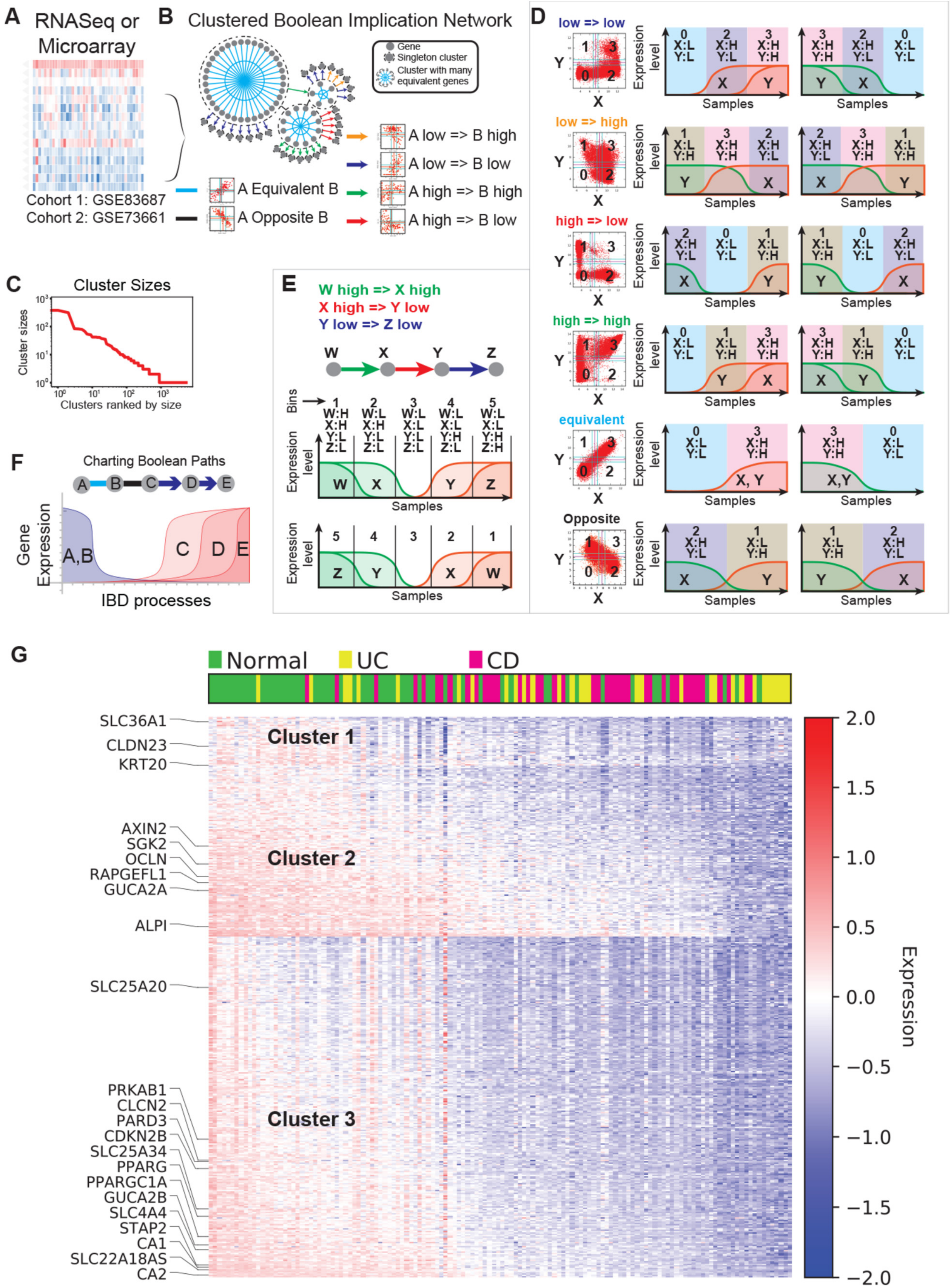

Fig. S1. Boolean Network Explorer (*BoNE*): A tool for clustering and visualization of Boolean implication network. (A) BoNE can be applied to any high-dimensional dataset. Here we applied *BoNE* to analyze IBD datasets: GSE83687 and GSE73661. **(B)** *BooleanNet* algorithm is applied to identify Boolean implication relationships. BoNE uses Boolean equivalent relationships to cluster genes and identify relationships between clusters. **(C)** The distribution of cluster sizes shows the expected scale-free architecture. A graphical display of cluster size analysis shows a linear trend in log-log scatterplots between clusters sorted by size and the number of clusters of any particular size. **(D)** Visualization of Boolean Implication relationships by one-dimensional plots of gene expression levels assuming gene expression value change (up or down) only once along a path. Samples are ordered in two possible ways to show the relationship. X low => Y low can be represented in a single dimension in two possible ways: (1) X turns on first and then Y turns on along a hypothetical biological path defined by the sample order. The path begins with X low and Y low, then it transitions to X high and Y low, and finally it transitions to X high and Y high. (2) Y turns off first and then X turns off along a hypothetical biological path defined by the sample order. The path begins with X high and Y high, then it transitions to X high and Y low; finally it transitions to both low. Similarly, the other Boolean relationships follow two possible path scenarios based on logical conclusions. **(E)** Visualization of a complex Boolean path using one-dimensional plot. (1) Three Boolean relationships between W, X, Y, and Z. (2) Graph representation of the Boolean relationships. Edges are colored differently according to the type of Boolean relationship. (3) W turns off first and then X turns off along a hypothetical biological path defined by the sample order followed by Y turning on and Z turning on. The path begins with W high, X high, Y low and Z low. (4) The path begins with W low, X low, Y high and Z high. Z turns off first and then Y turns off along a hypothetical biological path defined by the sample order followed by X turning on and W turning on. **(F)** Similar to panel E, four Boolean implication relationships A equivalent B, B opposite C, C low => D low, D low => E low constitute a Boolean path that can be used to develop a computational model of IBD biological process. The computational model predicts how these five genes might be changing along a biological path as shown in the plot. **(G)** Analysis of the IBD datasets using BoNE reveals a major Boolean path between clusters C1-2-3. Genes present in clusters 1, 2, and 3 is used to compute a score based on the weighted linear combination of normalized expression values. The score is used to order the samples from left to right. Heatmap of the gene expression values using genes present in clusters 1, 2, and 3 is computed. Key genes in the clusters are presented on the left of the heatmap. The expression level ranged from -2 to +2; the expression below 0 indicated low expression and represented with blue color and the expression above 0 indicated high expression and represented with red color.

A

Cohort	GSE#	# of Samples (N, IBD)	ROC AUC	Accuracy	Fisher Exact p value
Test 1	GSE83687	(60, 74)	0.81	0.75	5.13 e-09
Test 2	GSE73661	(12, 166)	0.98	0.94	8.11 e-10
Test 3	GSE6731	(4, 12)	1.00	0.94	0.003
Validation 1	GSE75214	(11, 82)	0.99	0.95	1.30 e-09
Validation 2	GSE16879	(6, 43)	1.00	1.00	7.15 e-08
Validation 3	GSE59071	(11, 105)	0.97	0.96	5.48 e-08
Validation 4	GSE48958	(8, 13)	0.91	0.76	0.007
Validation 5	GSE37283	(5, 15)	0.71	0.5	0.26
Validation 6	GSE109142	(20, 206)	0.97	0.95	7.23 e-12

B

Test Cohort 1: Peters et al. 2017; GSE83687

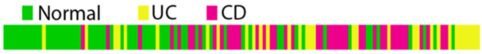

	Precesion	Recall	F1-score	Sample #
Normal (training)	0.72	0.73	0.73	60
IBD (training)	0.78	0.77	0.78	74

Test Cohort 2 : Arjis et al. 2018; GSE73661

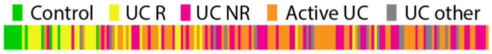

	Precesion	Recall	F1-score	Sample #
Normal (training)	0.38	0.93	0.54	42
IBD (training)	0.96	0.54	0.69	136

Test Cohort 3 : Wu et al. 2017; GSE6731

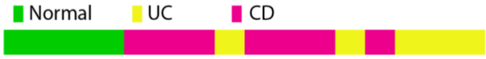

	Precesion	Recall	F1-score	Sample #
Normal	0.57	1.0	0.73	4
IBD	1.0	0.75	0.86	12

Validation Cohort 1: Vancamelbeke et al. 2017; GSE75214

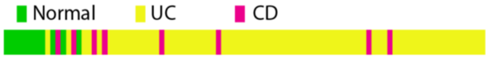

	Precesion	Recall	F1-score	Sample #
Normal	0.31	1.0	0.47	11
IBD	1.0	0.70	0.82	82

Validation Cohort 2: Arjis et al. 2009; GSE16879

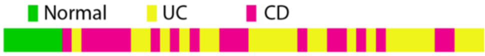

	Precesion	Recall	F1-score	Sample #
Normal	0.33	1.0	0.50	6
IBD	1.0	0.72	0.84	43

Validation Cohort 3: Vanhove et al. 2015; GSE59071

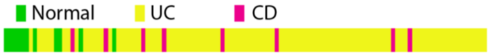

	Precesion	Recall	F1-score	Sample #
Normal	0.88	0.64	0.74	11
IBD	0.96	0.99	0.98	105

Validation Cohort 4: Van der Goten et al. 2014; GSE48958

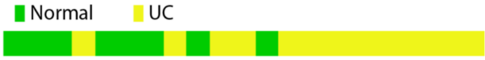

	Precesion	Recall	F1-score	Sample #
Normal	0.62	1.0	0.76	8
IBD	1.0	0.62	0.76	13

Validation Cohort 5: Pekow et al. 2013; GSE37283

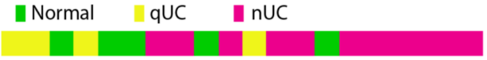

	Precesion	Recall	F1-score	Sample #
qUC	1.0	0.75	0.86	4
nUC	0.92	1.0	0.96	11

Validation Cohort 6: Denson et al. 2018; GSE109142

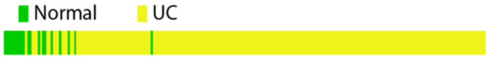

	Precesion	Recall	F1-score	Sample #
Normal	0.75	0.6	0.67	20
UC	0.96	0.98	0.97	206

Precesion= [TP/(TP+FP)]

Recall= [TP/(TP+FN)]

F1-score= [2 x (precision x recall)/(precision + recall)]

Fig. S2. Path C#1-2-3 predicts healthy vs disease samples. (A) BoNE identified path C1-2-3 by machine learning that performed best in separating healthy vs disease samples in three training datasets: GSE83687, GSE73661 and GSE6731. C1-2-3 path score is applied to six other validation datasets to predict healthy vs disease samples: GSE75214, GSE16879, GSE59071, GSE48958, GSE37283, and GSE109142. The strength of the classification is measured by the number of samples, ROC AUC, Accuracy, and Fisher exact p-values. Fisher exact test (two-sided) is performed on a 2x2 contingency table based on the prediction. **(B)** Detailed classification report is provided in terms of heatmap of the sample ordering, precision (TP/(TP+FP)), recall (TP/(TP+FN)), and f1-score (2 * (precision * recall)/(precision + recall)) for all the datasets.

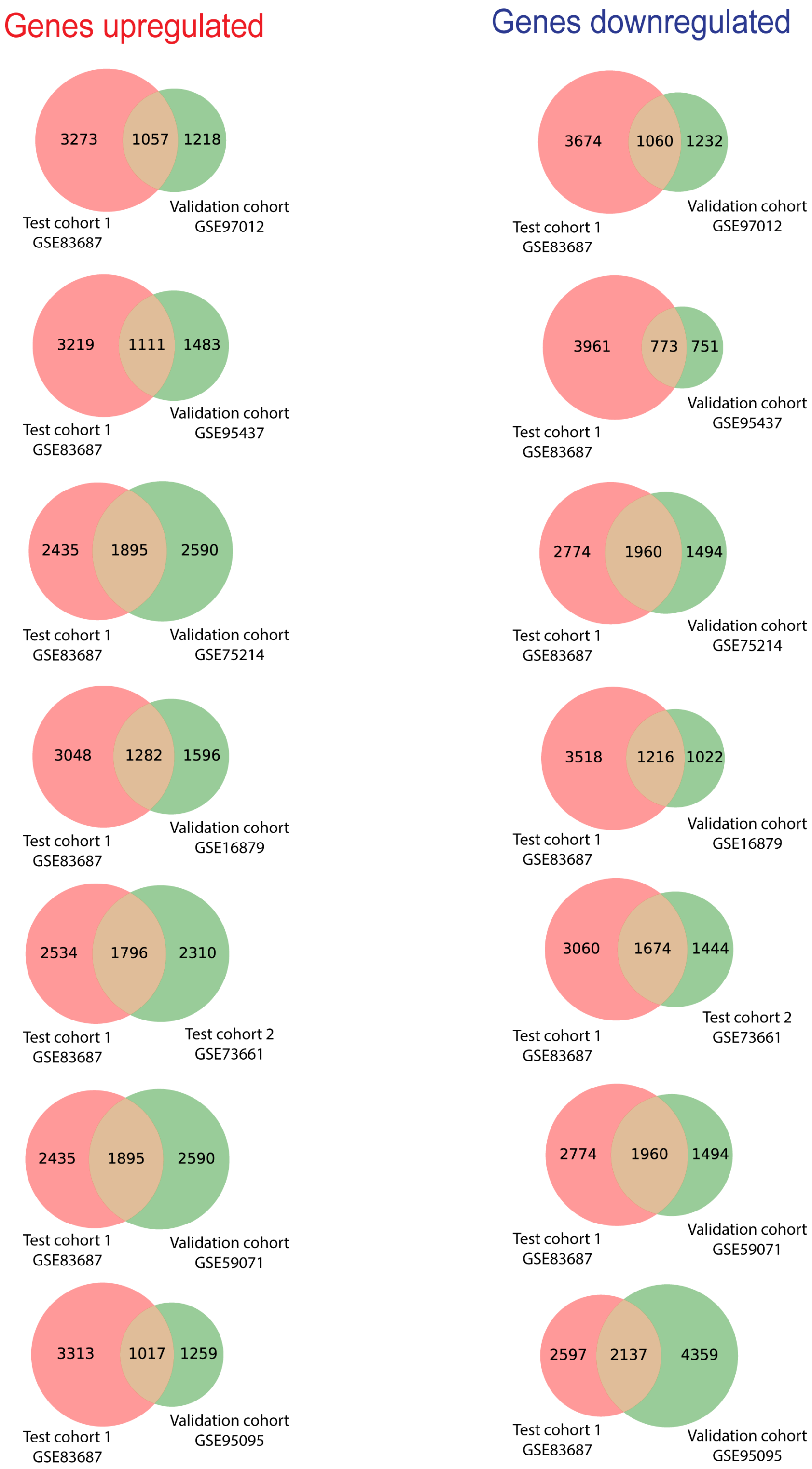

Fig. S3. Limited overlap of differentially expressed genes between two independent datasets: Test cohort 1 GSE83687 is compared to seven distinct independent IBD datasets: GSE97012, GSE95437, GSE75214, GSE16879, GSE73661, GSE59071, GSE95095. Overlap of up- and down-regulated genes are illustrated using two Venn diagrams.

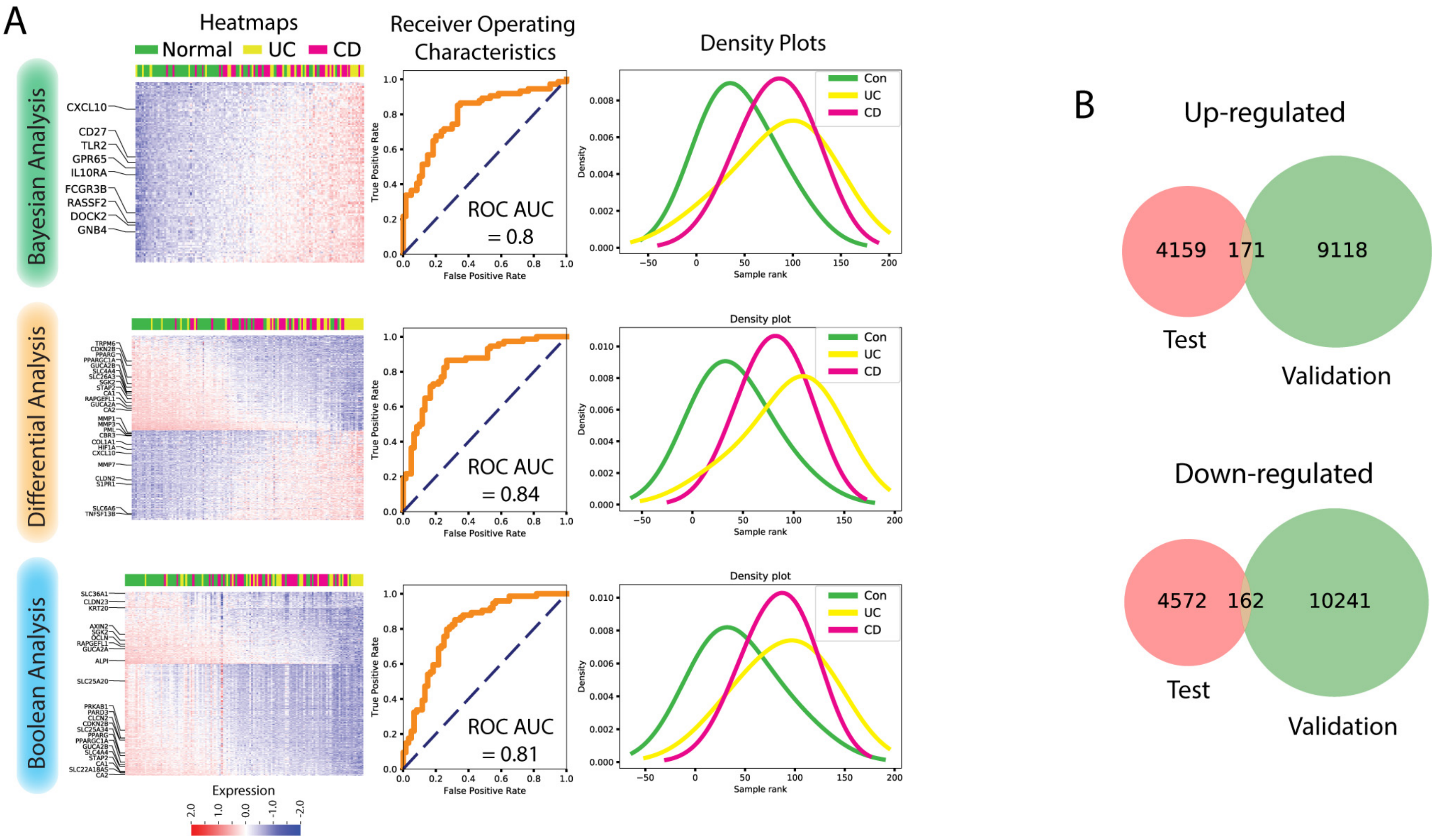

Fig. S4. Boolean, Differential and Bayesian analyses perform similarly regarding their ability to accurately distinguish healthy from diseased tissue samples: **(A)** Test cohort 1 GSE83687 is used to compare how well healthy and disease samples are separated by Boolean, Differential and Bayesian analysis. **(B)** Union of all genes that were differentially expressed is shown in Venn diagrams to have few overlaps for both up- and down-regulated genes.

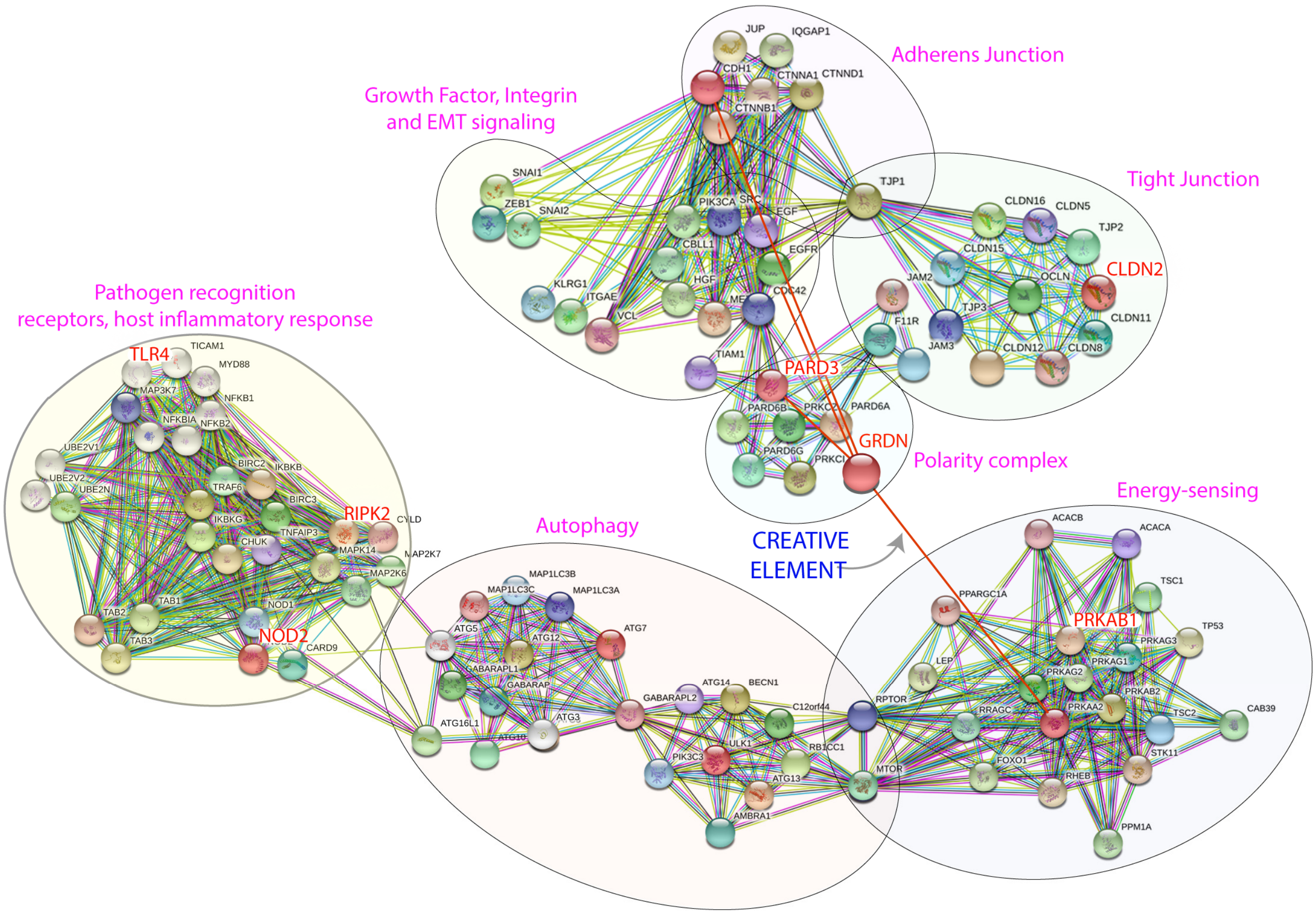

Fig S5. Protein-protein interaction network reveals that the stress-polarity signaling (SPS)-pathway is a creative element within that network: Protein-protein interaction (PPI) network built using STRING software (https://string-db.org/) shows the major modules and inter-module links between pathogen-sensing pathways (left most) to epithelial cell-cell adhesions (top right). A stress polarity signaling (SPS) pathway which involves the phosphorylation of the polarity scaffold, Girdin (GRDN), by the metabolic kinase AMPK (of which PRKAB1 is a subunit) has been described as both necessary and sufficient for the strengthening of epithelial junctions under bioenergetic stress (*24*). Because this event is triggered exclusively as a stress response and helps connect distinct modules of PPI, it fulfills the criteria of "creative elements" within this network (*35*).

A

SNP	BP position	[a]Reference allele	Reported Allele frequency	[b]Best phenotype	[c]LR phenotype	[d]Log-10 BF	[e]Het I2	Eur. O .R	Eur. P	Candidate Genes	Ref. [PMID]
rs11064881	120146925	A	7.34%	IBD	IBD_U	7.02	31.7	1.1 (1.06-1.14)	5.95×10^{-8}	PRKAB1	26192919

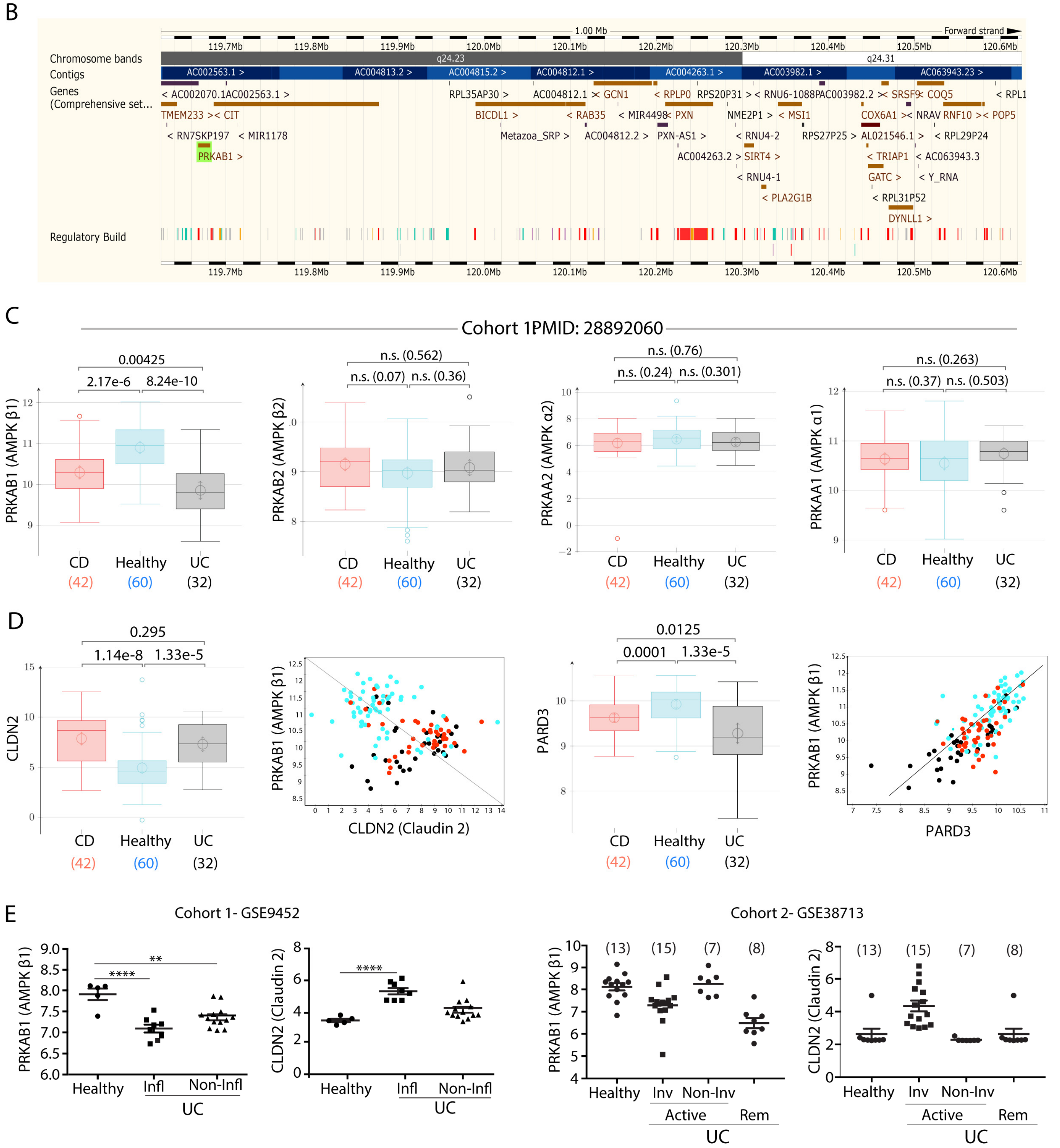

Fig S6. Transcripts of PRKAB1, but not other PRKA/PRKB subunits, is selectively downregulated in IBD: (A) Table showing details of SNP reported in patients with IBD. [a]The minor allele in the European cohort was chosen to be the reference allele. [b]Phenotype with the largest MANTRA Bayes factor. [c]The preferred phenotype (Ulcerative Colitis, Crohn's Disease or IBD (i.e. both)) from our likelihood modeling approach to classify loci according to their relative strength of association. IBD_S and IBD_U refer

to the IBD saturated and IBD unsaturated models, respectively (*36*). [d]MANTRA log_{10} Bayes Factor. [e]Hetrogeneity I^2 percentage. **(B)** Genome browser view of PRKAB1 locus showing that the IBD-associated SNP in PRKAB1 is localized to non-coding regions. **(C)** Box plots showing analysis of publically available IBD transcriptomic datasets (*1*) identifying PRKAB1 as the only AMPK subunit dysregulated in UC and CD patients. The box in the box plot shows the interquartile range (IQR, distance between lower and upper quartile). From above the upper quartile, a distance of 1.5 times the IQR is measured out and a whisker is drawn up to the largest observed point from the dataset that falls within this distance. Similarly, a distance of 1.5 times the IQR is measured out below the lower quartile and a whisker is drawn up to the lower observed point from the dataset that falls within this distance. All other observed points are plotted as outliers. Center of the circle represent the average value and the length of arrows up/down represent 95% confidence interval. P values are computed using standard two-sided unequal variance (Welch) t-test. **(D)** Bar graphs and scatter plots showing PRKAB1 down regulation in IBD correlates with upregulation of CLND2 (leaky TJ protein) and down regulation of PARD3 (essential polarity protein). **(E)** Scatter plots showing PRKAB1 and CLDN2 dysregulation in IBD were confirmed using an independent transcriptomic dataset. Data are shown as mean ± S.E.M. and one-way ANOVA using Tukey's multiple comparisons test and $p \leq 0.05$ cutoff was used to determine significance;('*/**/***/***' represents p-values where; *; $p \leq 0.05$, **; $p \leq 0.01$, ***; $p \leq 0.001$, ****; $p \leq 0.0001$).

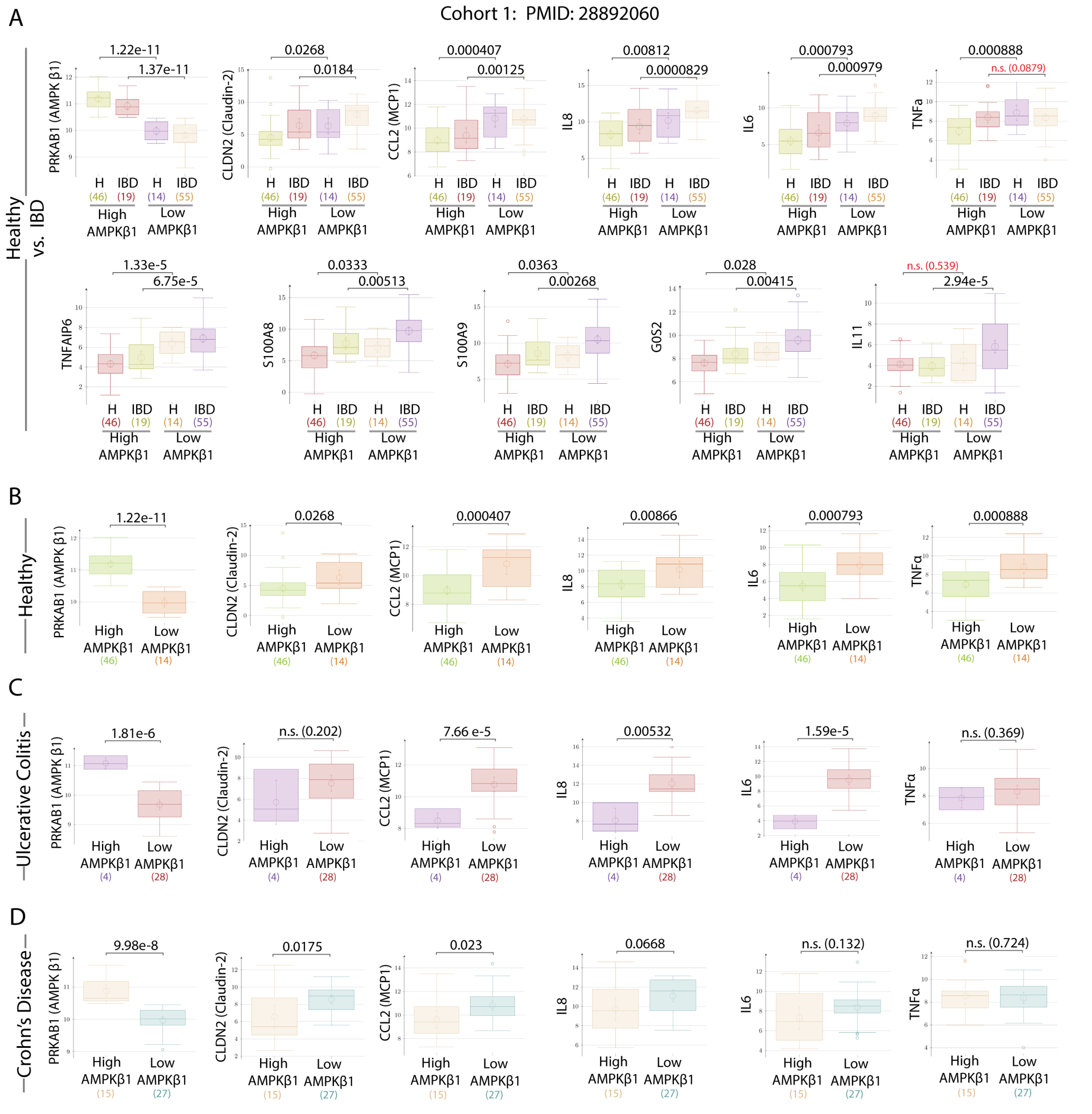

Fig S7. Gene expression analysis confirms that low levels of PRKAB1 correlate with a higher degree of leakiness of the epithelial barrier (CLDN2), proinflammatory cytokines (MCP1, IL8, IL6 and TNFα), and a signature for non-response to Infliximab (TNFAIP6, S100A8,, S100A9, IL-11, and GOS2(*37*)): Healthy and IBD patients were divided into two groups with high vs. low PRKAB1 mRNA levels using a statistically determined cut-off (*StepMiner* threshold; (*11*)). Box plots show the expression of various transcripts analyzed between the two groups in **(A)** pooled IBD patient samples, **(B)** Healthy Controls, **(C)** Ulcerative Colitis patients, and **(D)** Crohn's Disease patients. The box in the box plot shows the interquartile range (IQR, distance between lower and upper quartile). From above the upper quartile, a distance of 1.5 times the IQR is measured out and a whisker is drawn up to the largest observed point from the dataset that falls within this distance. Similarly, a distance of 1.5 times the IQR is measured out below the lower quartile and a whisker is drawn up to the lower observed point from the dataset that falls within this distance. All other observed points are plotted as outliers. Center of the circle represent the average value and the length of arrows up/down represent 95% confidence interval. P values are computed using standard two-sided unequal variance (Welch) t-test.

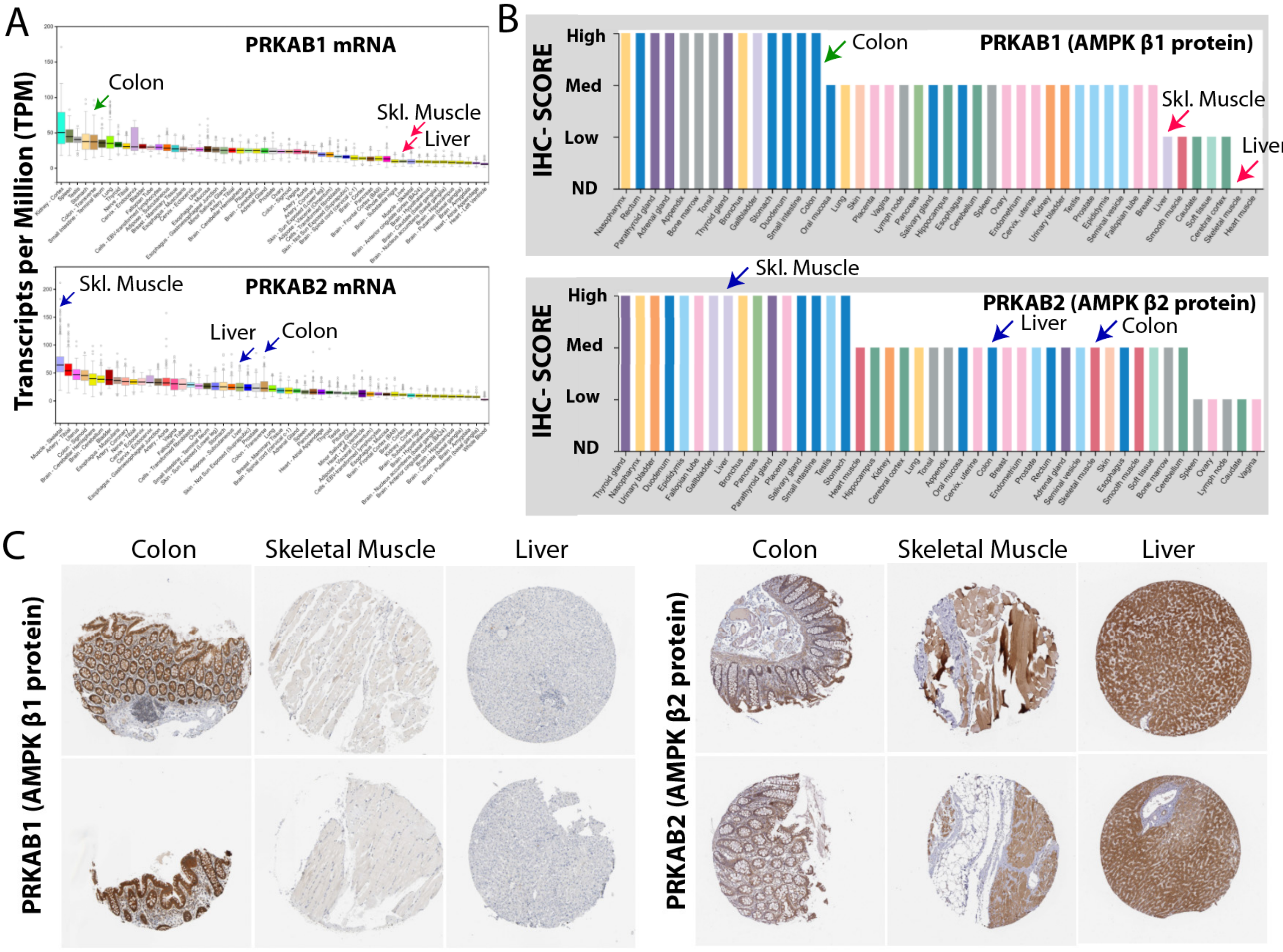

Fig S8. PRKAB1 mRNA and protein are preferentially expressed in the colon: (A) Boxplots showing PRKAB1 subunit of AMPK mRNA is expressed at high levels in the colon (**green arrow**), but low levels in liver and skeletal muscle (**red arrows**), where PRKAB2 subunit is expressed in all three tissues with the highest expression in skeletal muscle (**blue arrows**). The box in the box plot shows the interquartile range (IQR, distance between lower and upper quartile). From above the upper quartile, a distance of 1.5 times the IQR is measured out and a whisker is drawn up to the largest observed point from the dataset that falls within this distance. Similarly, a distance of 1.5 times the IQR is measured out below the lower quartile and a whisker is drawn up to the lower observed point from the dataset that falls within this distance. All other observed points are plotted as outliers. **(B)** Bar graph summarizing protein expression data determined by IHC on colon biopsies. PRKAB1 is highly expressed in the colon (**green arrow**) compared to liver and skeletal muscle (**red arrows**) compared to PRKAB2, which shows no preferential expression between tissues (**blue arrow**). **(C)** Representative tissue IHC images used for protein expression analysis. All data curated from Human Protein Atlas (www.proteinatlas.org).

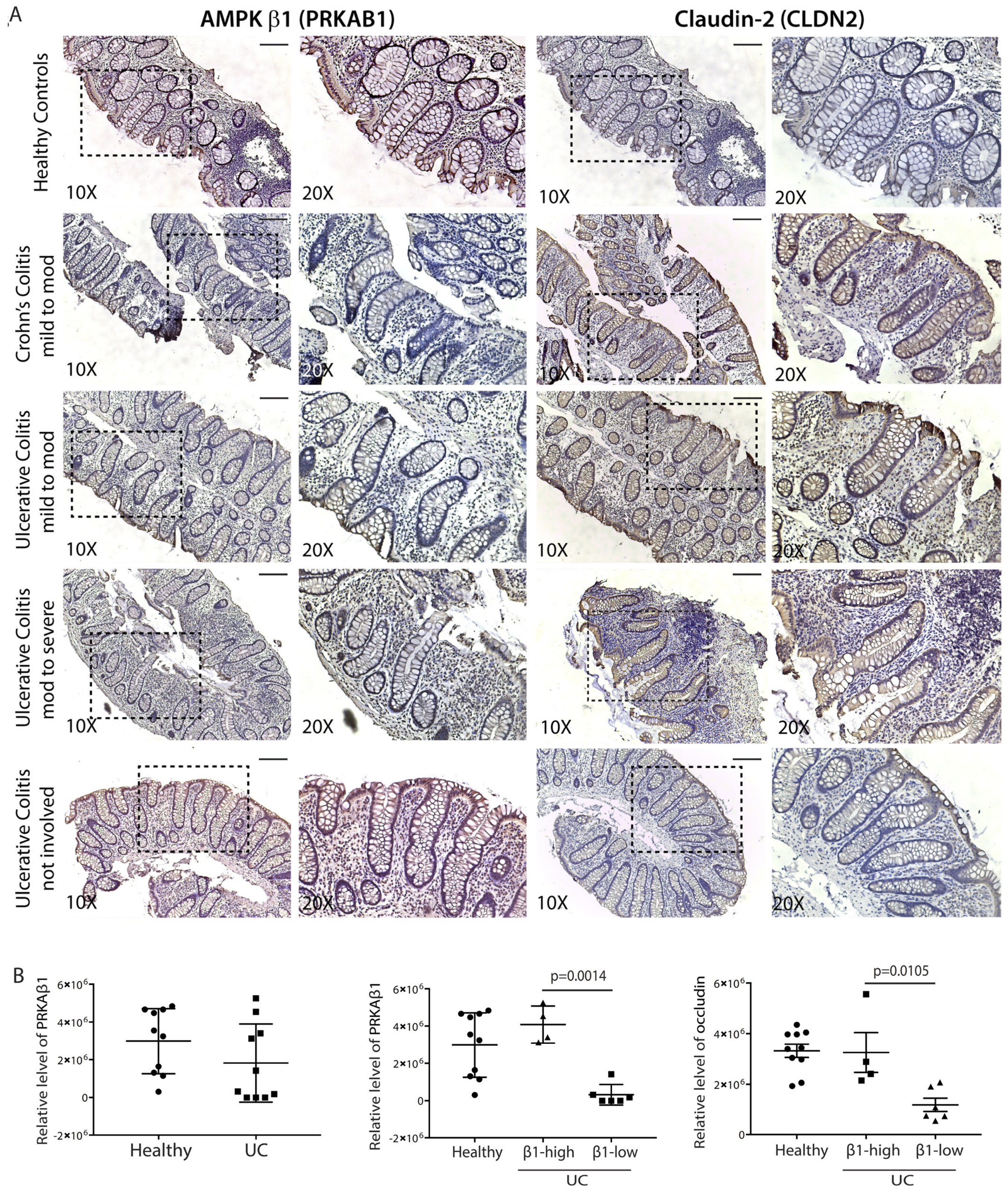

Fig S9. IHC of FFPE colon biopsies and colon-derived proteomics analysis confirms Boolean relationships of PRKAB1 at the protein level: (A) Expression of PRKAB1 and CLDN2 analyzed by IHC on FFPE IBD patient colon biopsies from various stages of disease severity. Representative images are shown. Boxed region represents the areas that are presented at 20X magnification. Scale bar = 100 µm. **(B)** Proteomic datasets (*32*) from healthy and UC patients were analyzed for PRKAB1 expression. Samples were sub-divided into PRKAB1 high vs. low and assessed for expression of the TJ protein occludin. Claudin-2 peptides were not detected reliably in this study, and hence, not analyzed. Data displayed as scatter plots showing mean ± S.E.M. and one-way ANOVA using Tukey's multiple comparisons test and $p \leq 0.05$ cutoff was used to determine significance.

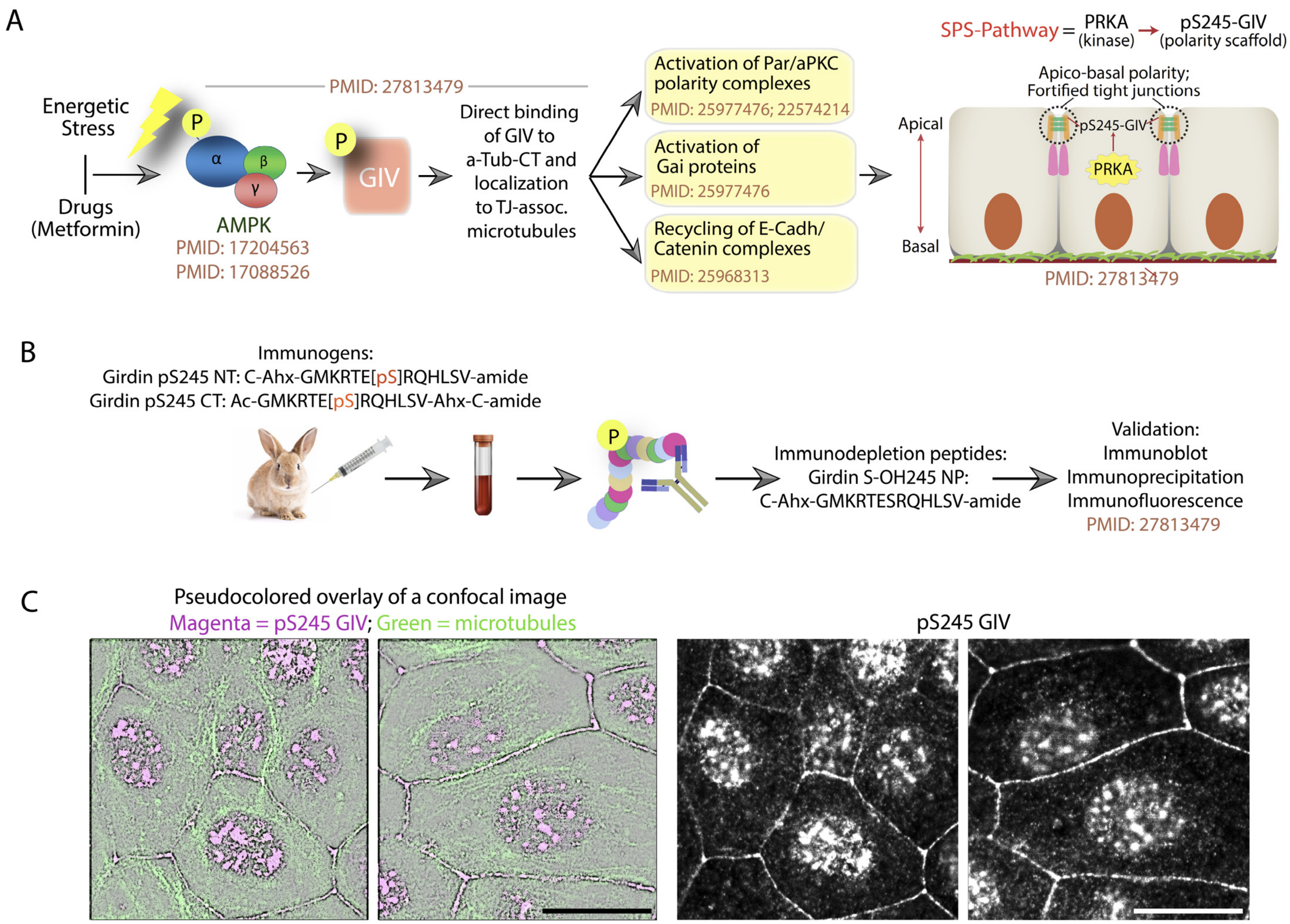

Fig S10. A method to detect the stress-polarity signaling (SPS)-pathway in cells: (A) Schematic describing the antigen used for generating a phospho-serine (S245) specific GIV/Girdin antibody used to monitor activation of SPS-pathway. Validation studies using this reagent are published elsewhere (*24*). **(B)** Selected immunofluorescent (IF) images showing localization of pS245-GIV to cell-cell junctions of MDCK cells. Intranuclear signal of unknown significance is noted. **(C)** MDCK cells were grown on glass cover slips, exposed or not to energetic stress (exactly as done previously (*24*) via glucose deprivation for 120 min) and subsequently fixed and stained for phospho (p) GIV (magenta) and tubulin (green) and analyzed by confocal microscopy. Representative confocal images are shown. Scale bar = 10 µm.

A

Compounds	Chemical names	Selectivity	Structural basis for MOA	AC50 or EC50		Barrier protective effect in colitis
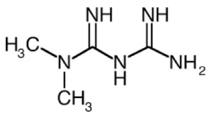 Metformin	N,N-Dimethylimidodicarbonimidic diamide	Non-selective indirect AMPK activator; via AMP	AMP binding site PDB: 4RED	Surrogate [AMP] PMID: 26635351	Activation AC50 α1β1γ1 (3064 nM) α2β1γ1 (471 nM) α1β2γ1 (1415 nM) α2β2γ1 (129 nM)	Tested; protects in DSS colitis (2.5%), at dose100-150 mg/kg
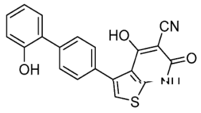 A-769662	6, 7 Dihydro-4-hydroxy-3-(2′-hydroxyl [1,1′-biphenyl]-4-yl) -6-oxo-thieno[2,3-b] pyridone-5-carbonitrile	β1-selective AMPK activator	molecule-bound structure PDB: 4CFF	PMID: 26635351	Activation AC50 α1β1γ1 (72.24 nM) α2β1γ1 (1415 nM) α1β2γ1 (> 40,000 nM) α2β2γ1 (>40,000 nM)	Not tested
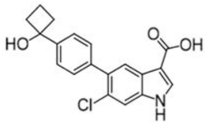 PF-06409577	6-Chloro-5-[4-(1-hydroxycyclobutyl) phenyl]-1H-indole-3-carboxylic acid	β1-selective AMPK activator	molecule-bound structure PDB: 5T5T	PMID: 27490827	Activation EC50 α1β1γ1 (8.2 nM) α2β1γ1 (6.8 nM) α1β2γ1 (> 40,000 nM) α2β2γ1 (>40,000 nM)	Not tested

B

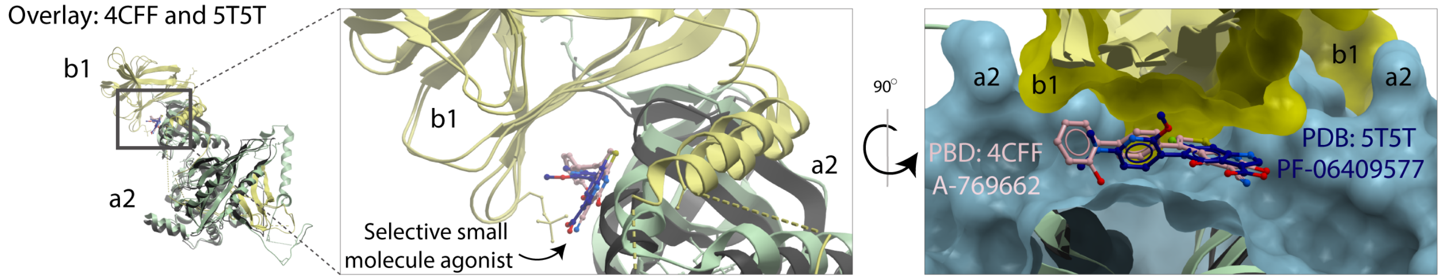

Fig S11. Pharmacologic properties of β1- selective AMPK agonists: (A) Table outlining pharmacologic properties of AMPK agonists used. **(B)** Structural model of AMPKβ1 and α2 interface highlighting the binding pocket of β1-selective AMPK agonists.

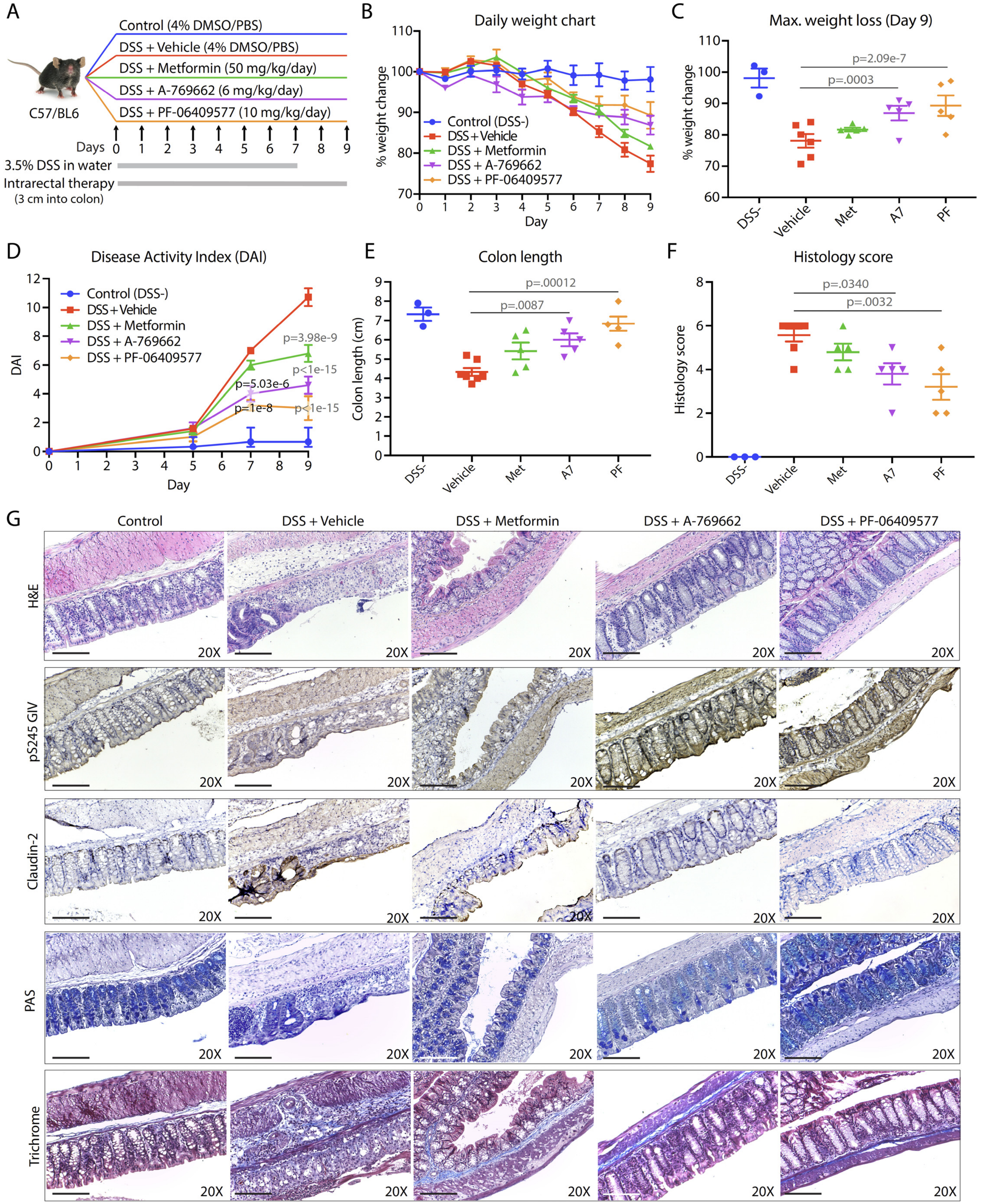
A
Control (4% DMSO/PBS)
DSS + Vehicle (4% DMSO/PBS)
DSS + Metformin (50 mg/kg/day)
DSS + A-769662 (6 mg/kg/day)
DSS + PF-06409577 (10 mg/kg/day)
C57/BL6
Days 0 1 2 3 4 5 6 7 8 9
3.5% DSS in water
Intrarectal therapy
(3 cm into colon)
B
Daily weight chart
% weight change
Day
Control (DSS-)
DSS+Vehicle
DSS + Metformin
DSS + A-769662
DSS + PF-06409577
C
Max. weight loss (Day 9)
% weight change
p=2.09e-7
p=.0003
DSS-
Vehicle
Met
A7
PF
D
Disease Activity Index (DAI)
DAI
Day
Control (DSS-)
DSS+Vehicle
DSS + Metformin
DSS + A-769662
DSS + PF-06409577
p=3.98e-9
p<1e-15
p=5.03e-6
p=1e-8
p<1e-15
E
Colon length
Colon length (cm)
p=.00012
p=.0087
F
Histology score
Histology score
p=.0340
p=.0032
G
Control
DSS + Vehicle
DSS + Metformin
DSS + A-769662
DSS + PF-06409577
H&E
pS245 GIV
Claudin-2
PAS
Trichrome
20X

Fig S12. Extended dataset demonstrating the efficacy of β1-selective AMPK agonists in an acute DSS colitis: (A) Schematic was outlining **the** experimental design of DSS colitis model used to test the efficacy of PRKAB1 agonists. **(B)** Line graph showing body weight change monitored daily during acute DSS colitis. **(C)** Scatter plot showing maximum weight loss (observed on d9) in DSS experiment. **(D)** Line graph of disease activity index (DAI) scored using stool consistency (0-4), rectal bleeding (0-4), and weight loss (0-4). **(E)** Scatter plot of colon length assessed at d9 of DSS experiment. **(F)** Scatter plot of histomorphological evaluation of inflammation by hematoxylin and eosin (H&E) stained colon tissues using inflammatory cell infiltrate (1-3), and epithelial architecture (1-3) as scoring criteria. For D-F, n=3-7 mice per group (DSS, n=3; Vehicle, n=7; Met, n=5; A7, n=5; PF, n=5) **(G)** Representative images of colon tissue stained with H&E, or immunostained for activation of SPS-pathway (pS245 GIV) or upregulation of Claudin-2. Tissues were also stained to assess goblet cells loss (PAS staining) and fibrotic collagen deposition (Trichrome stain). All scale bars are 200 µm. All data are shown as mean ± S.E.M. and one (C, E, F) or two-way (B, D) ANOVA using Tukey's multiple comparisons test and $p \leq 0.05$ cutoff was used to determine significance.

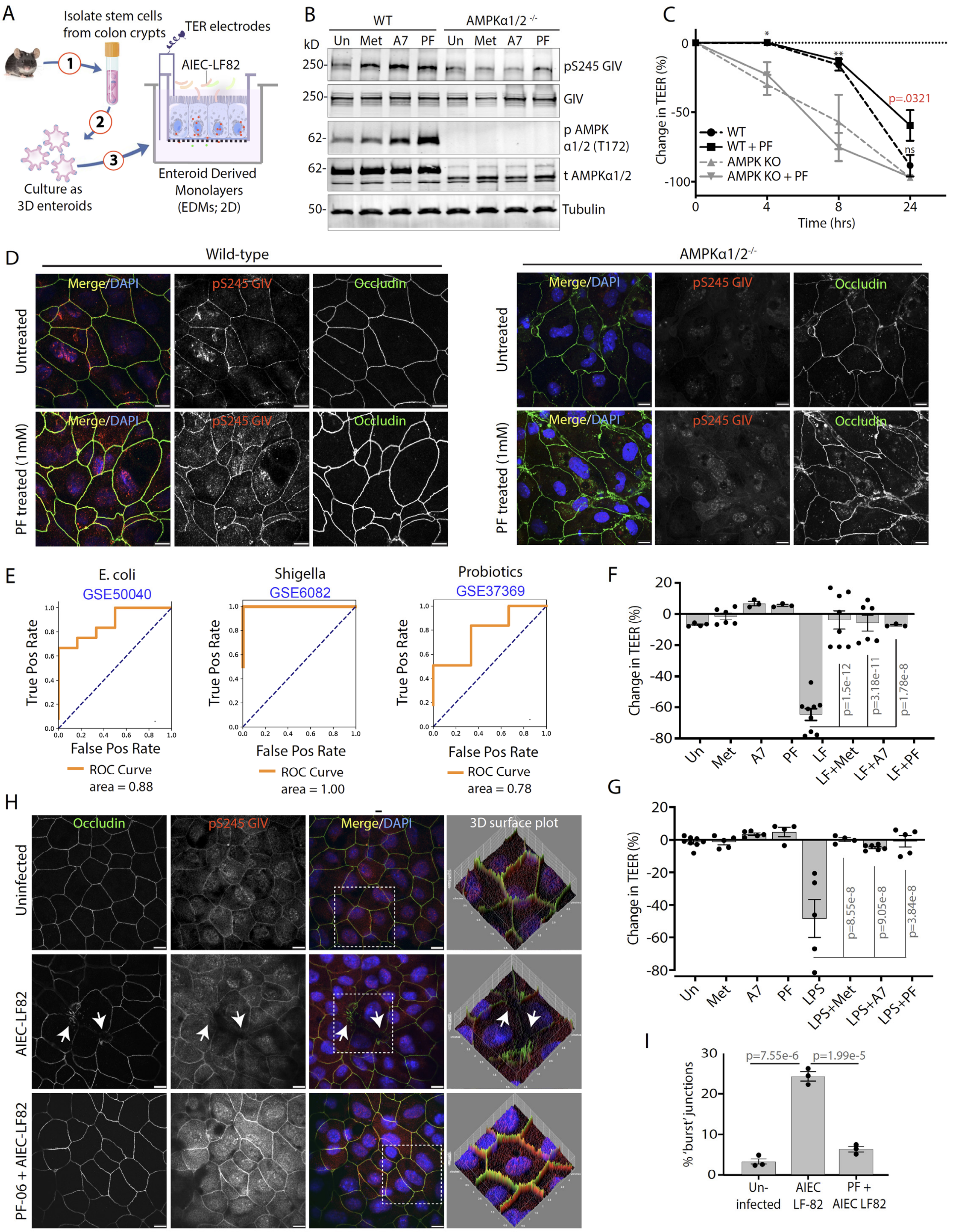
A
Isolate stem cells
from colon crypts
TER electrodes
AIEC-LF82
1
2
3
Culture as
3D enteroids
Enteroid Derived
Monolayers
(EDMs; 2D)
B
WT
AMPKα1/2 -/-
kD
Un Met A7 PF Un Met A7 PF
250-
pS245 GIV
250-
GIV
62-
p AMPK
α1/2 (T172)
62-
t AMPKα1/2
50-
Tubulin
C
Change in TEER (%)
0
-50
-100
*
**
p=.0321
ns
WT
WT + PF
AMPK KO
AMPK KO + PF
0 4 8 24
Time (hrs)
D
Wild-type
AMPKα1/2-/-
Merge/DAPI
pS245 GIV
Occludin
Untreated
PF treated (1mM)
E
E. coli
GSE50040
Shigella
GSE6082
Probiotics
GSE37369
True Pos Rate
False Pos Rate
ROC Curve
area = 0.88
ROC Curve
area = 1.00
ROC Curve
area = 0.78
F
Change in TEER (%)
20
0
-20
-40
-60
-80
p=1.5e-12
p=3.18e-11
p=1.78e-8
Un Met A7 PF LF LF+Met LF+A7 LF+PF
G
Change in TEER (%)
p=8.55e-8
p=9.05e-8
p=3.84e-8
Un Met A7 PF LPS LPS+Met LPS+A7 LPS+PF
H
Occludin
pS245 GIV
Merge/DAPI
3D surface plot
Uninfected
AIEC-LF82
PF-06 + AIEC-LF82
I
% 'burst' junctions
30
20
10
0
p=7.55e-6
p=1.99e-5
Un-
infected
AIEC
LF-82
PF +
AIEC LF82

Fig S13. Murine colon-derived organoid monolayers confirm that pharmacologic activation of PRKAB1 protects the epithelial barrier by IBD-associated microbes: (A) Schematic of the stem cell-based organoid model and generation of enteroid-derived monolayers (EDMs). **(B)** Immunoblots of 3D enteroid whole cell lysates isolated from WT or AMPK KO mice treated with pharmacologic agonists of AMPK for 4 h (1 mM Metformin; 100 µM A-769662; 10 µM PF-06409557). **(C)** Line graph showing TEER kinetics over 24 h of WT or AMPKα1/2$^{-/-}$ EDM pre-treated or not with PF for 16 h. Black asterisks (*) = WT vs. AMPK KO comparisons; red asterisks (*) = untreated vs. PF pre-treatment. n=4 per biological replicates per group. **(D)** EDMs treated as in (C) were assessed for TJ integrity (occludin) and SPS-pathway activation (pS245 GIV) using confocal microscopy at 24h. All scale bars are 10 µm. **(E)** ROC/AUC plots rationalizing the use of monolayer-microbe co-culture systems to model pathologic shifts. (**F-G)** Bar graphs showing the change in TEER in EDMs pre-treated with indicated AMPK agonists [Met = metformin. A7 = A-769662; PF = PF-06409557] and subsequently exposed to adherent invasive *E. coli* (*AIEC*-LF82 strain) **(F)**, or LPS **(G)** at 8 h post-infection. **(H)** EDMs pre-treated with indicated AMPK agonists for 16 h were assessed for TJ integrity (occludin) and SPS-pathway activation (pS245 GIV) after exposure to adherent invasive *E. coli* (*AIEC*-LF82 strain) for 8 h using confocal microscopy. Images are representative fields of 3 independent experiments. All scale bars are 10 µm. **(I)** Bar graph quantifying the frequency of burst TJs observed in EDMs treated as in **(F)**. Quantification was done on 3 randomly chosen fields in each of three independent experiments. All data displayed as mean ± S.E.M. and one-way ANOVA using Tukey's multiple comparisons test and $p \leq 0.05$ cutoff was used to determine significance.

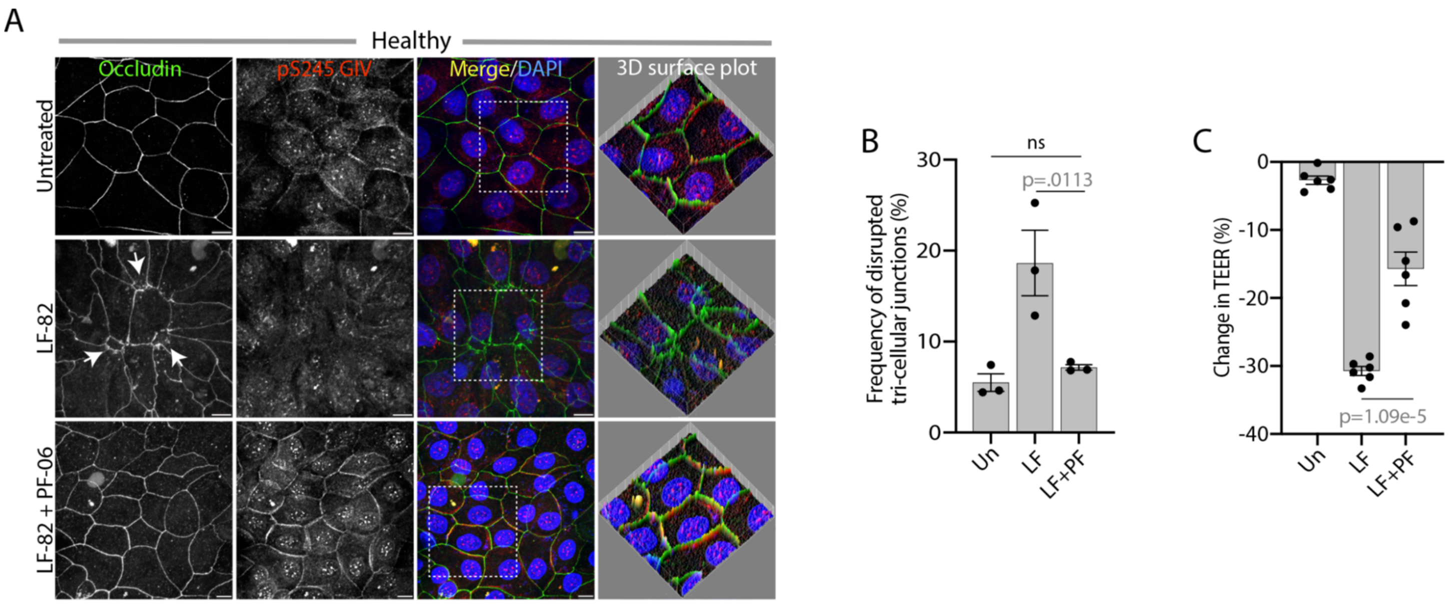

Fig S14. PRKAB1 agonists protect healthy human enteroid-derived monolayers from microbe-induced barrier collapse. **(A)** Healthy human EDM pre-treated, or not, with PF for 16 h were assessed for TJ integrity (occludin) and SPS-pathway activation (pS245 GIV) after exposure to adherent invasive *E. coli* (*AIEC*; LF-82 strain) for 8h using confocal microscopy. White arrowheads identify disrupted TJs. The overlap of pS245-GIV and occludin pixels after AMPK activation is visualized as 3D surface plots. Images are representative fields of 3 independent experiments. All scale bars are 10 µm. **(B)** Bar graph showing quantification of disrupted tricellular tight junctions (TTJs) in healthy human EDMs treated as in (D). **(C)** Bar graph showing change in TEER of healthy human EDMs treated or not with AMPKβ1-agonist followed by challenge with *AIEC*-LF-82. LF = *AIEC*-LF-82. Data are shown as mean ± S.E.M. and one-way ANOVA using Tukey's multiple comparisons test and $p \leq 0.05$ cutoff was used to determine significance.

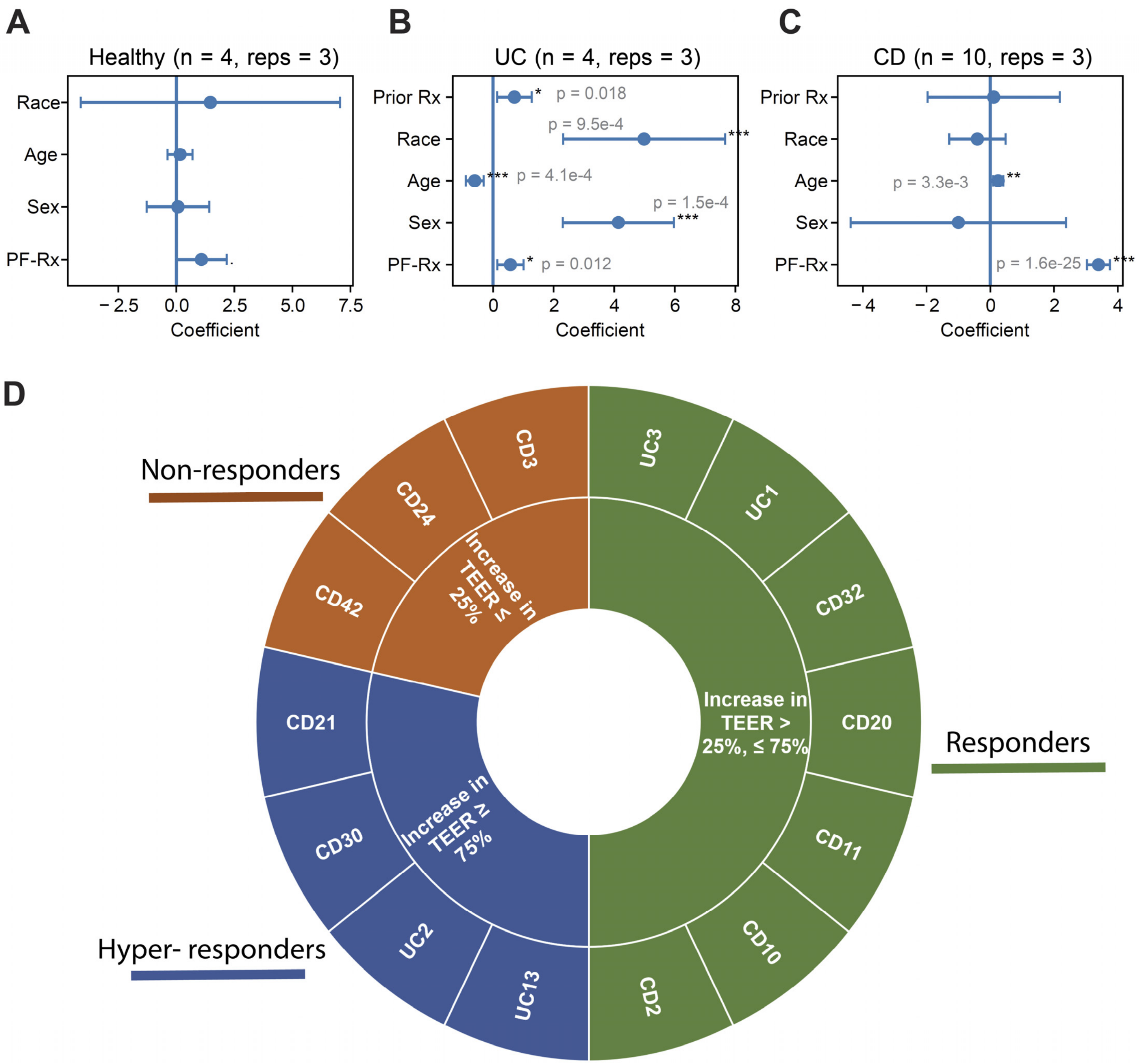

Fig S15. Treatment with PRKAB1-agonist (PF) is an independent indicator of response (increase in TEER) in IBD patient-derived organoids. Multivariate analysis models the TEER measurement before the treatment as a linear combination of TEER measurement after treatment, age, gender (Female:0, Male:1), race (African American:0, Asian:1, Caucasian:2, Hispanic:3, Middle Eastern:4), treatment history (Naive:0, Infliximab:1, Adalimumab:2, Adalimumab + Infliximab:4, Vedolizumab:3, Vedolizumab + Adalimumab:5). Coefficient of each variables (at the center) with 95% confidence intervals (as error bars) and the *p*-values were illustrated in the bar plot for (A-C). The p-value for each term tests the null hypothesis that the coefficient is equal to zero (no effect). **(A)** Healthy (n = 4, average 3 repeats per experiment), **(B)** UC (n = 3, average 3 repeats per experiment), and **(C)** CD (n = 9, average 3 repeats per experiment). Significance based on different cutoffs is denoted using: *; $p \leq 0.05$, **; $p \leq 0.01$, ***; $p \leq 0.001$, ****; $p \leq 0.0001$. **(D)** The pie chart displays the proportion and type of disease subtype and their response to treatment with PRKABI-agonist PF (PF-Rx). CD = Crohn's disease; UC = Ulcerative colitis. See **Table 2** for details on cohort composition.

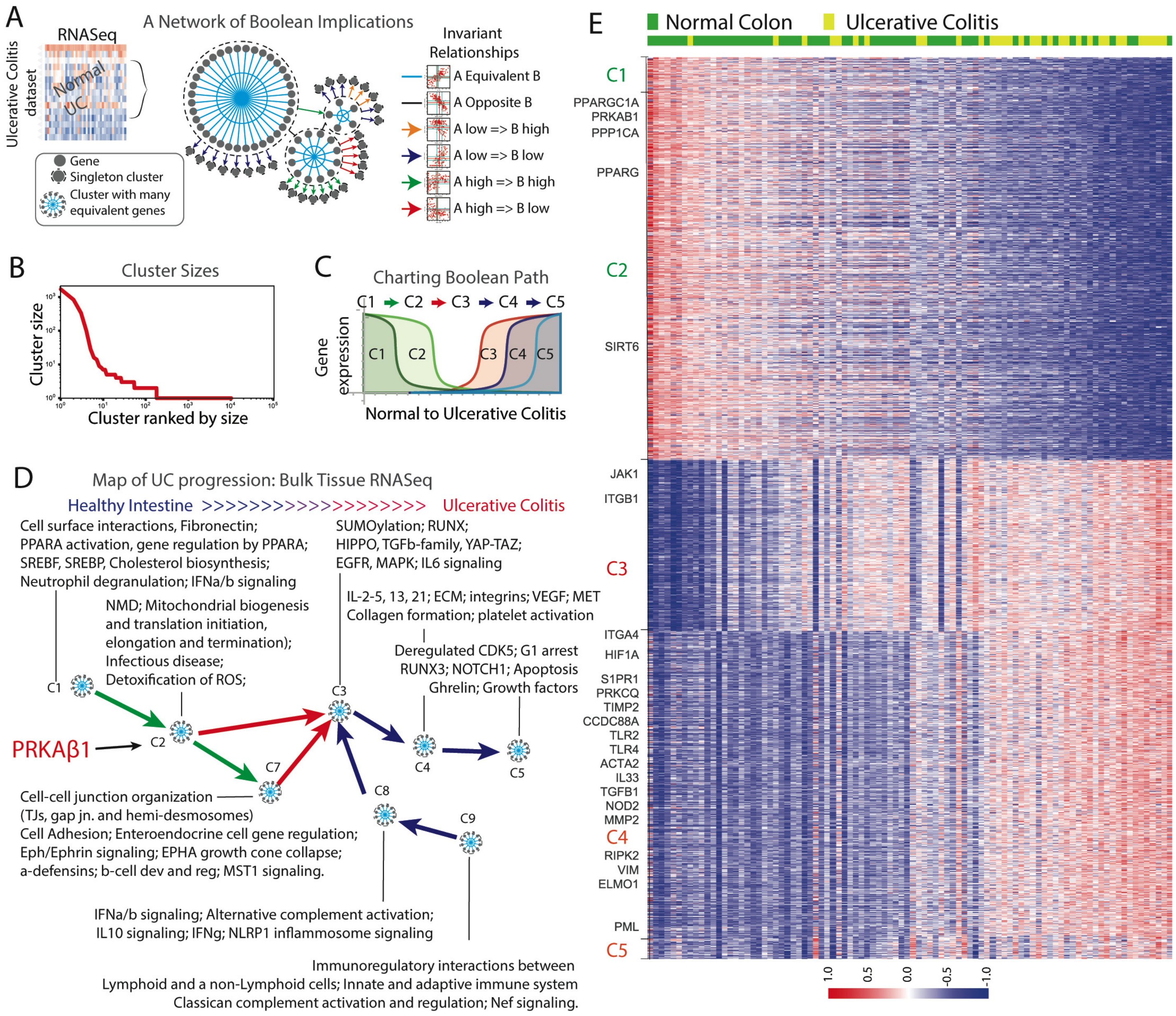

Fig S16. Boolean implication network and disease map of Ulcerative Colitis: (A) Boolean network analysis was performed on pooled Ulcerative colitis transcriptomic datasets (*1*) to identify global pathways and biological processes enriched in a continuum of cellular states during UC progression. The Boolean network (middle) contains the six possible Boolean relationships between genes (right) in the form of a directed graph with nodes as genes and edges as relationships between genes. Genes with similar expression profiles were organized into clusters, and relationships between clusters represented as color-coded edges connecting clusters. **(B)** Graph displaying the distribution of sizes of gene clusters that are equivalent to each other confirms scale-free architecture of the Boolean network in A. **(C)** Schematic showing individual gene expression changes along a boolean path within the normal to UC continuum, illustrating the changing levels of expression of the genes in the above example. **(D)** Reactome pathway analysis of each clusters along the top continuum paths were performed to identify the signaling pathways and cellular processes that are enriched during UC progression (PRKAB1 is within cluster #2). **(E)** Heatmap of the expression profile of genes within Boolean clusters superimposed on sample type (top bar) shows the accuracy of Boolean analysis in sample segregation into normal and UC. See **Supplementary Data 3** for cluster composition and detailed reactome pathway analyses.

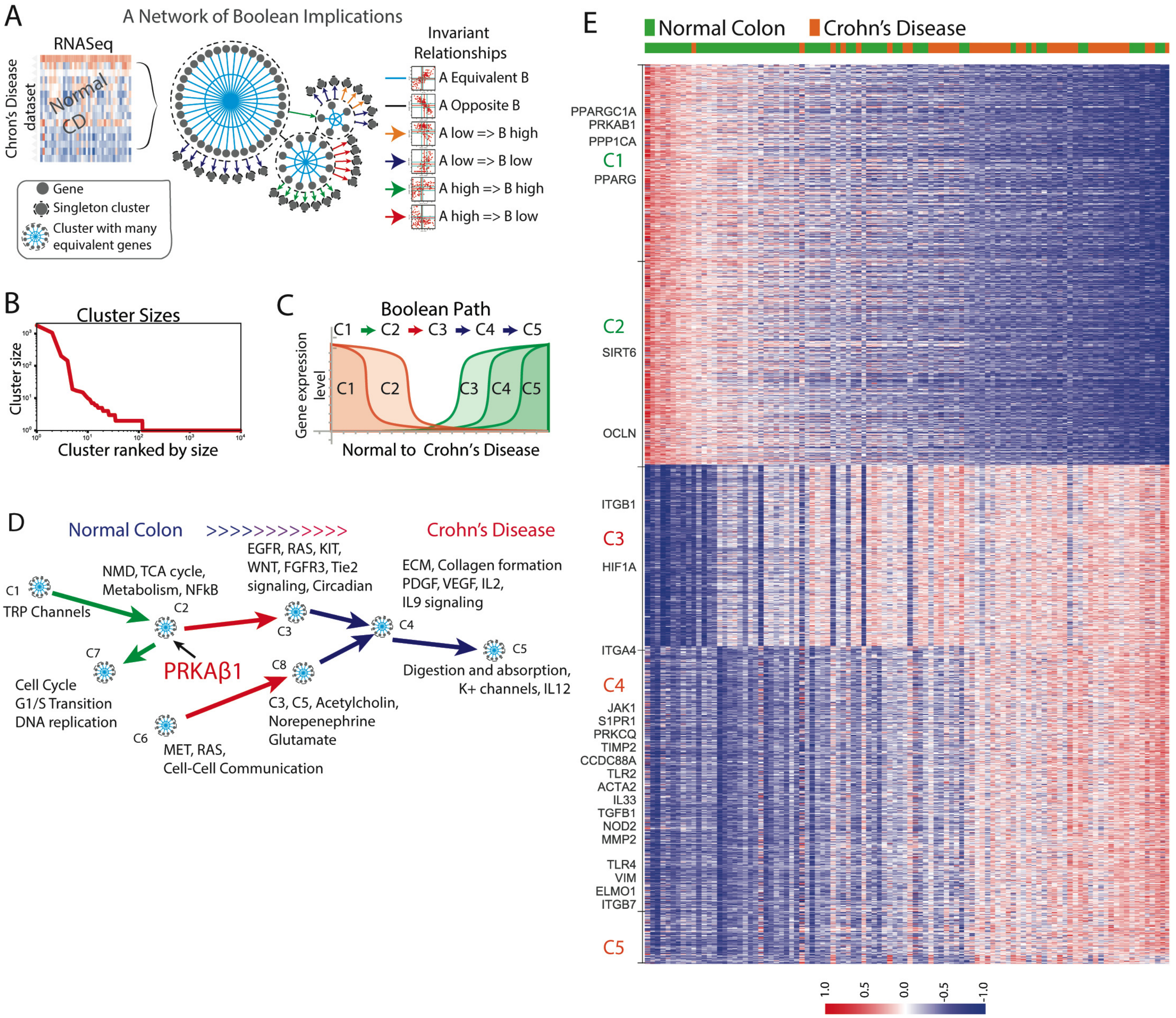

Fig S17. Boolean implication network and disease map of Crohn's Disease: (A) Boolean network analysis was performed on pooled Crohn's disease transcriptomic datasets (*1*) to identify global pathways and biological processes enriched in a continuum of cellular states during CD progression. The Boolean network (middle) contains the six possible Boolean relationships between genes (right) in the form of a directed graph with nodes as genes and edges as relationships between genes. Genes with similar expression profiles were organized into clusters, and relationships between clusters represented as color-coded edges connecting clusters. **(B)** Graph displaying the distribution of sizes of gene clusters that are equivalent to each other confirms scale-free architecture of the Boolean network in A. **(C)** Schematic showing individual gene expression changes along a boolean path within the normal to CD continuum, illustrating the changing levels of expression of the genes in the above example. **(D)** Reactome pathway analysis of each clusters along the top continuum paths were performed to identify the signaling pathways and cellular processes that are enriched during CD progression (PRKAB1 is within cluster #2). **(E)** Heatmap of the expression profile of genes within Boolean clusters superimposed on sample type (top bar) shows the accuracy of Boolean analysis in sample segregation into normal and CD. See **Supplementary Data 4** for cluster composition and detailed reactome pathway analyses.

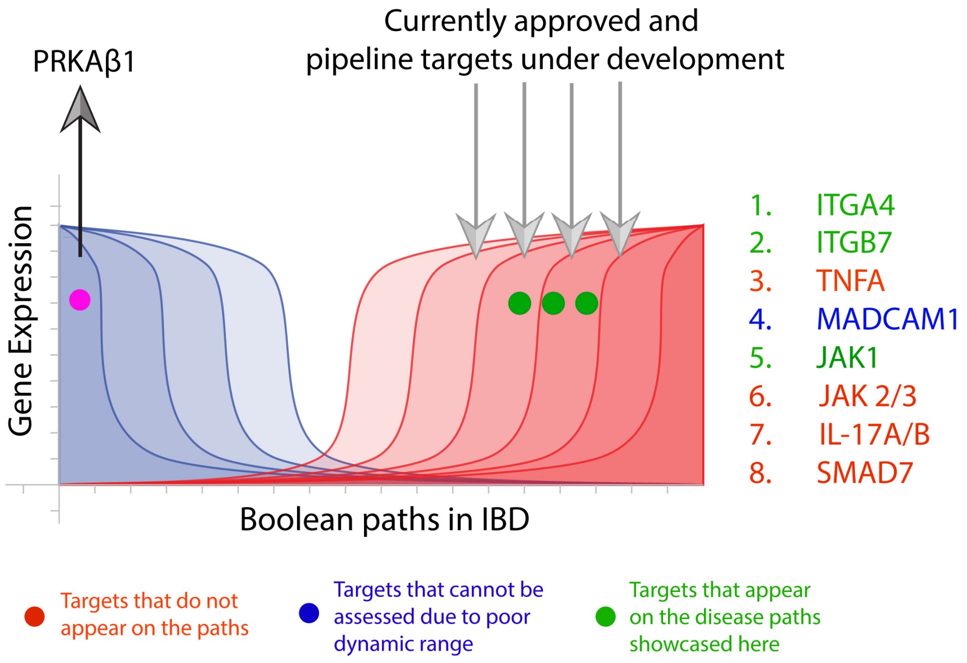

Fig S18. PRKAB1-agonists are predicted to augment healthy gut barrier function; combination with anti-inflammatory agents is predicted to show therapeutic synergy: PRKAB1 and a few other selected targets currently approved, or in development for treating IBD are superimposed on Boolean Network path model. Targets either do not appear (Red), and unable to be assessed (Blue), or reside on the 'disease' end (Green) of IBD progression spectrum. PRKAB1 is positioned on the 'healthy' end of the network, where pharmacologic augmentation is predicted to restore or protect mucosal homeostasis and barrier function. Combination of PRKAB1-agonists (enhancers of the healthy network) with anti-inflammatory agents (suppressors of diseased genes) is predicted to show therapeutic synergy.

A

	Boolean					Differential					Bayesian					Support [Total # targets]
	Precision	Recall	F1-score	Acc-uracy	Fisher's p- value	Precision	Recall	F1-score	Acc-uracy	Fisher's p- value	Precision	Recall	F1-score	Acc-uracy	Fisher's p- value	
Failure	1.00	1.00	1.00			0.00	0.00	0.00			1.00	1.00	1.00			16
Success	1.00	1.00	1.00	1.00	4.9141e-05	0.24	1.00	0.38	0.238	1.0	1.00	1.00	1.00	0.4761	0.2621	5
Avg./Total	1.00	1.00	1.00			0.06	0.24	0.09			0.51	0.48	0.49			21

Precision [TP/(TP + FP)]; Recall [TP/(TP+FN)]; F1-score [2 x (precision x recall)/(precision + recall)]
TP = True Positives; FP= False positives; FN = False negatives

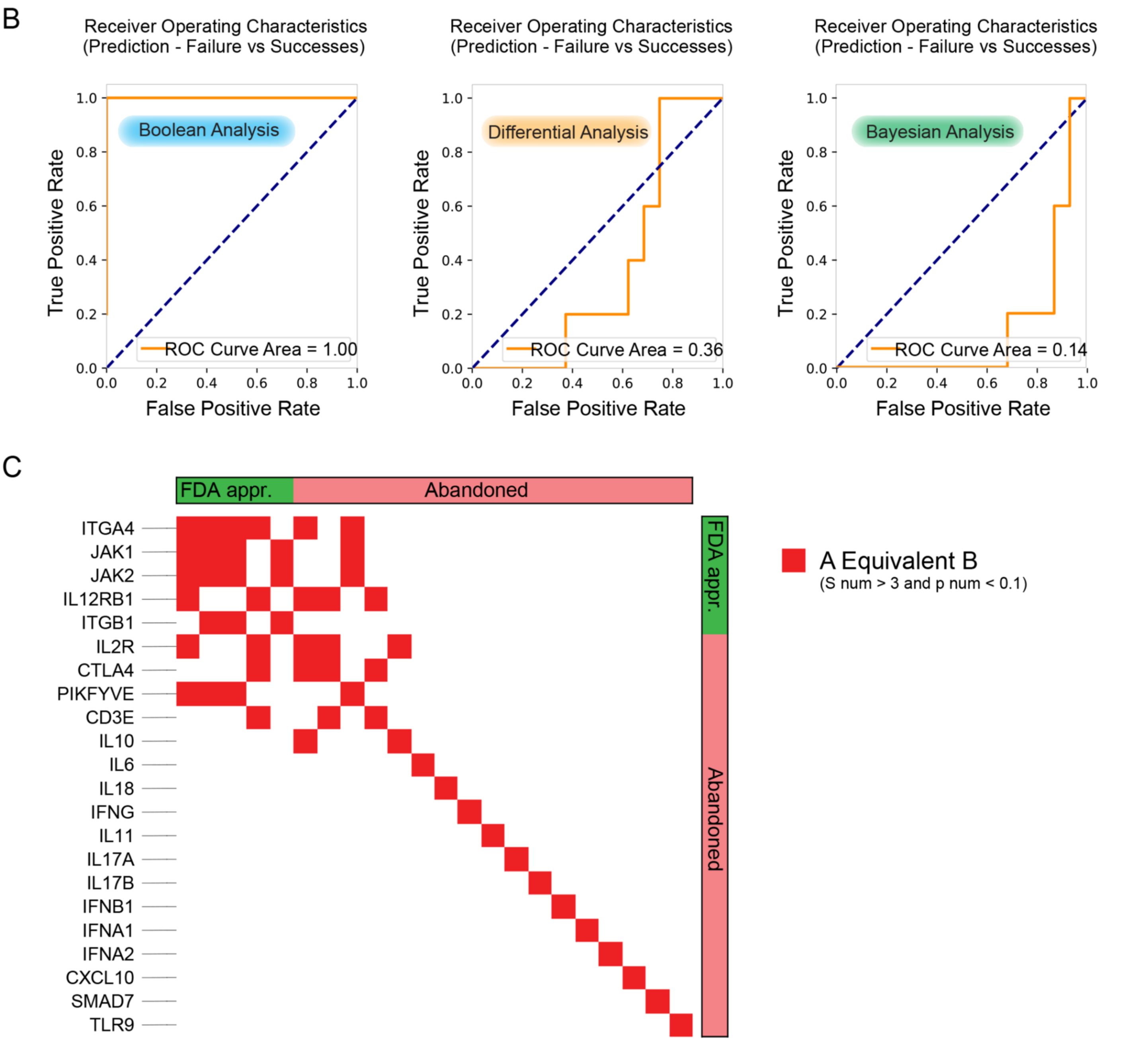

Fig S19. Prediction of outcome of Phase III clinical trials by Differential, Bayesian and Boolean analyses. **(A)** Prediction accuracy measured by precision, recall, f1-score of three different computational approaches (Differential, Bayesian and Boolean) compared to the known clinical trial outcomes of 21 targets [**Table 3**]. Fisher exact test (two-sided) is performed on a 2x2 contingency table based on the prediction. **(B)** ROC (receiver operating characteristic) curve and ROC-AUC (area under the curve) is presented in the plot for all three computational approaches: Differential, Bayesian and Boolean. (C) Boolean Equivalent relationships (in GSE83687, using BooleanNet statistical test (*9*)) between FDA-approved and abandoned targets. FDA-approved targets tend to implicate each other using the Boolean Equivalent relationship.

Tables (Uploaded individually as Word Document Tables)

Table 1: *Genes that share a strong Boolean implication relationship with PRKAB1 on the major continuum paths in IBD (related to* ***Fig 3C-D****).*

Table 2. *Cohort characteristics for human organoid-based studies [related to Fig 4H-R, 5A, Main Text]*

Table 3. *Statistical analysis of the likelihood of success (FDA approval) of targets in IBD [related to* ***Fig 5C-D****].*

Supplementary Files (Uploaded individually as Supplementary Datasets)

Supplementary Data 1. *Transcriptomic datasets analyzed in this work and the heterogeneity of samples in those datasets.*

Supplementary Data 2. *Gene clusters on the IBD map (**Fig 2**) and the corresponding Reactome pathway analyses of each cluster.*

Supplementary Data 3. *Gene clusters on the UC-alone map (**Fig S16**) and the corresponding Reactome pathway analyses of each cluster.*

Supplementary Data 4. *Gene clusters on the CD-alone map (**Fig S17**) and the corresponding Reactome pathway analyses of each cluster.*

References.

1. L. A. Peters *et al.*, A functional genomics predictive network model identifies regulators of inflammatory bowel disease. *Nat Genet* **49**, 1437-1449 (2017).
2. I. Arijs *et al.*, Effect of vedolizumab (anti-alpha4beta7-integrin) therapy on histological healing and mucosal gene expression in patients with UC. *Gut* **67**, 43-52 (2018).
3. R. Edgar, M. Domrachev, A. E. Lash, Gene Expression Omnibus: NCBI gene expression and hybridization array data repository. *Nucleic Acids Res* **30**, 207-210 (2002).
4. T. Barrett *et al.*, NCBI GEO: archive for functional genomics data sets--update. *Nucleic Acids Res* **41**, D991-995 (2013).
5. T. Barrett *et al.*, NCBI GEO: mining tens of millions of expression profiles--database and tools update. *Nucleic Acids Res* **35**, D760-765 (2007).
6. P. Dalerba *et al.*, Single-cell dissection of transcriptional heterogeneity in human colon tumors. *Nat Biotechnol* **29**, 1120-1127 (2011).
7. P. Dalerba *et al.*, CDX2 as a Prognostic Biomarker in Stage II and Stage III Colon Cancer. *N Engl J Med* **374**, 211-222 (2016).
8. J. P. Volkmer *et al.*, Three differentiation states risk-stratify bladder cancer into distinct subtypes. *Proc Natl Acad Sci U S A* **109**, 2078-2083 (2012).
9. D. Sahoo, D. L. Dill, A. J. Gentles, R. Tibshirani, S. K. Plevritis, Boolean implication networks derived from large scale, whole genome microarray datasets. *Genome Biol* **9**, R157 (2008).
10. D. Sahoo *et al.*, MiDReG: a method of mining developmentally regulated genes using Boolean implications. *Proc Natl Acad Sci U S A* **107**, 5732-5737 (2010).
11. D. Sahoo, D. L. Dill, R. Tibshirani, S. K. Plevritis, Extracting binary signals from microarray time-course data. *Nucleic Acids Res* **35**, 3705-3712 (2007).
12. I. Arijs *et al.*, Mucosal gene expression of antimicrobial peptides in inflammatory bowel disease before and after first infliximab treatment. *PLoS One* **4**, e7984 (2009).
13. M. A. Inlay *et al.*, Ly6d marks the earliest stage of B-cell specification and identifies the branchpoint between B-cell and T-cell development. *Genes Dev* **23**, 2376-2381 (2009).
14. D. Sahoo *et al.*, Boolean analysis identifies CD38 as a biomarker of aggressive localized prostate cancer. *Oncotarget* **9**, 6550-6561 (2018).
15. A. Fabregat *et al.*, The Reactome Pathway Knowledgebase. *Nucleic Acids Res* **46**, D649-D655 (2018).
16. L. Chen *et al.*, Activating AMPK to Restore Tight Junction Assembly in Intestinal Epithelium and to Attenuate Experimental Colitis by Metformin. *Front Pharmacol* **9**, 761 (2018).
17. A. Bai *et al.*, AMPK agonist downregulates innate and adaptive immune responses in TNBS-induced murine acute and relapsing colitis. *Biochem Pharmacol* **80**, 1708-1717 (2010).
18. M. Takahara *et al.*, Berberine improved experimental chronic colitis by regulating interferon-gamma- and IL-17A-producing lamina propria CD4(+) T cells through AMPK activation. *Sci Rep* **9**, 11934 (2019).
19. B. Xu *et al.*, Geniposide ameliorates TNBS-induced experimental colitis in rats via reducing inflammatory cytokine release and restoring impaired intestinal barrier function. *Acta Pharmacol Sin* **38**, 688-698 (2017).
20. S. J. Koh, J. M. Kim, I. K. Kim, S. H. Ko, J. S. Kim, Anti-inflammatory mechanism of metformin and its effects in intestinal inflammation and colitis-associated colon cancer. *J Gastroenterol Hepatol* **29**, 502-510 (2014).
21. Y. Xue, H. Zhang, X. Sun, M. J. Zhu, Metformin Improves Ileal Epithelial Barrier Function in Interleukin-10 Deficient Mice. *PLoS One* **11**, e0168670 (2016).
22. J. Blagih *et al.*, The energy sensor AMPK regulates T cell metabolic adaptation and effector responses in vivo. *Immunity* **42**, 41-54 (2015).

23. H. Le-Niculescu, I. Niesman, T. Fischer, L. DeVries, M. G. Farquhar, Identification and characterization of GIV, a novel Galpha i/s-interacting protein found on COPI, endoplasmic reticulum-Golgi transport vesicles. *The Journal of biological chemistry* **280**, 22012-22020 (2005).
24. N. Aznar *et al.*, AMP-activated protein kinase fortifies epithelial tight junctions during energetic stress via its effector GIV/Girdin. *Elife* **5**, (2016).
25. M. M. Mahe, N. Sundaram, C. L. Watson, N. F. Shroyer, M. A. Helmrath, Establishment of human epithelial enteroids and colonoids from whole tissue and biopsy. *J Vis Exp*, (2015).
26. T. Sato *et al.*, Single Lgr5 stem cells build crypt-villus structures in vitro without a mesenchymal niche. *Nature* **459**, 262-265 (2009).
27. H. Miyoshi, T. S. Stappenbeck, In vitro expansion and genetic modification of gastrointestinal stem cells in spheroid culture. *Nat Protoc* **8**, 2471-2482 (2013).
28. G. den Hartog *et al.*, Regulation of Rac1 and Reactive Oxygen Species Production in Response to Infection of Gastrointestinal Epithelia. *PLoS Pathog* **12**, e1005382 (2016).
29. A. Darfeuille-Michaud *et al.*, High prevalence of adherent-invasive Escherichia coli associated with ileal mucosa in Crohn's disease. *Gastroenterology* **127**, 412-421 (2004).
30. K. Suarez *et al.*, Dysregulation of the engulfment pathway in the gut fuels Inflammatory Bowel Disease. *BIORXIV* **280172**, (2018).
31. P. Ghosh, M. Garcia-Marcos, S. J. Bornheimer, M. G. Farquhar, Activation of Galphai3 triggers cell migration via regulation of GIV. *The Journal of cell biology* **182**, 381-393 (2008).
32. T. B. Bennike *et al.*, Neutrophil Extracellular Traps in Ulcerative Colitis: A Proteome Analysis of Intestinal Biopsies. *Inflamm Bowel Dis* **21**, 2052-2067 (2015).
33. J. J. Kim, M. S. Shajib, M. M. Manocha, W. I. Khan, Investigating intestinal inflammation in DSS-induced model of IBD. *J Vis Exp*, (2012).
34. S. Das *et al.*, ELMO1 has an essential role in the internalization of Salmonella Typhimurium into enteric macrophages that impacts disease outcome. *Cell Mol Gastroenterol Hepatol* **1**, 311-324 (2015).
35. P. Csermely, Creative elements: network-based predictions of active centres in proteins and cellular and social networks. *Trends Biochem Sci* **33**, 569-576 (2008).
36. J. Z. Liu *et al.*, Association analyses identify 38 susceptibility loci for inflammatory bowel disease and highlight shared genetic risk across populations. *Nat Genet* **47**, 979-986 (2015).
37. I. Arijs *et al.*, Predictive value of epithelial gene expression profiles for response to infliximab in Crohn's disease. *Inflamm Bowel Dis* **16**, 2090-2098 (2010).
