# Supplementary material for: Artificial intelligence guided discovery of a barrier-protective therapy in inflammatory bowel disease
Source: Nat Commun. 2021 Jul 12;12:4246. doi: 10.1038/s41467-021-24470-5 (PMC8275683; doi:10.1038/s41467-021-24470-5)
Supplement: Supplementary file 3 — Description of Additional Supplementary Files [file 41467_2021_24470_MOESM3_ESM.docx]

Description of Additional Supplementary Files

**Supplementary Data 1.** *Transcriptomic datasets analyzed in this work* *and the heterogeneity of samples in those datasets*

**Supplementary Data 2.** *Gene clusters on the IBD map (****Fig 2****) and the corresponding Reactome pathway analyses of each cluster.*

**Supplementary Data 3.** *Gene clusters on the UC-alone map (****Fig S16****) and the corresponding Reactome pathway analyses of each cluster.*

**Supplementary Data 4.** *Gene clusters on the CD-alone map (****Fig S17****) and the corresponding Reactome pathway analyses of each cluster.*
