# Supplementary material for: Artificial intelligence guided discovery of a barrier-protective therapy in inflammatory bowel disease
Source: Nat Commun. 2021 Jul 12;12:4246. doi: 10.1038/s41467-021-24470-5 (PMC8275683; doi:10.1038/s41467-021-24470-5)
Supplement: Supplementary file 8 — Reporting Summary [file 41467_2021_24470_MOESM8_ESM.pdf]

nature research

Corresponding author(s): Debashis Sahoo, Soumita Das, Pradipta Ghosh

Last updated by author(s): 06/01/2021

Reporting Summary

Nature Research wishes to improve the reproducibility of the work that we publish. This form provides structure for consistency and transparency in reporting. For further information on Nature Research policies, see our Editorial Policies and the Editorial Policy Checklist.

Statistics

For all statistical analyses, confirm that the following items are present in the figure legend, table legend, main text, or Methods section.

n/a	Confirmed	
☐	☒	The exact sample size (n) for each experimental group/condition, given as a discrete number and unit of measurement
☐	☒	A statement on whether measurements were taken from distinct samples or whether the same sample was measured repeatedly
☐	☒	The statistical test(s) used AND whether they are one- or two-sided<br>*Only common tests should be described solely by name; describe more complex techniques in the Methods section.*
☐	☒	A description of all covariates tested
☐	☒	A description of any assumptions or corrections, such as tests of normality and adjustment for multiple comparisons
☐	☒	A full description of the statistical parameters including central tendency (e.g. means) or other basic estimates (e.g. regression coefficient) AND variation (e.g. standard deviation) or associated estimates of uncertainty (e.g. confidence intervals)
☐	☒	For null hypothesis testing, the test statistic (e.g. F, t, r) with confidence intervals, effect sizes, degrees of freedom and P value noted<br>*Give P values as exact values whenever suitable.*
☒	☐	For Bayesian analysis, information on the choice of priors and Markov chain Monte Carlo settings
☒	☐	For hierarchical and complex designs, identification of the appropriate level for tests and full reporting of outcomes
☒	☐	Estimates of effect sizes (e.g. Cohen's d, Pearson's r), indicating how they were calculated

Our web collection on statistics for biologists contains articles on many of the points above.

Software and code

Policy information about availability of computer code

Data collection	All datasets are publicly available in NCBI Gene Expression Omnibus.
Data analysis	Instructions for how to analyze dataset is available at https://github.com/sahoo00/BoNE. GraphPad Prism v9, ImageJ v1.53g, NCBI Primer Blast.

For manuscripts utilizing custom algorithms or software that are central to the research but not yet described in published literature, software must be made available to editors and reviewers. We strongly encourage code deposition in a community repository (e.g. GitHub). See the Nature Research guidelines for submitting code & software for further information.

Data

Policy information about availability of data

All manuscripts must include a data availability statement. This statement should provide the following information, where applicable:

- Accession codes, unique identifiers, or web links for publicly available datasets
- A list of figures that have associated raw data
- A description of any restrictions on data availability

All datasets are publicly available in Gene Expression Omnibus.

Field-specific reporting

Life sciences study design

All studies must disclose on these points even when the disclosure is negative.

Sample size	All available samples were used.
Data exclusions	No data were excluded from the analysis.
Replication	At least three reliable and successful biological replicates were used in each experiments.
Randomization	Sample allocation were controlled based on disease subtype. Healthy, UC and CD samples were used.
Blinding	Blinding was not possible. Investigator knew the disease status of each samples.

Reporting for specific materials, systems and methods

We require information from authors about some types of materials, experimental systems and methods used in many studies. Here, indicate whether each material, system or method listed is relevant to your study. If you are not sure if a list item applies to your research, read the appropriate section before selecting a response.

Materials & experimental systems

n/a	Involved in the study	
☐	☒	Antibodies
☐	☒	Eukaryotic cell lines
☒	☐	Palaeontology and archaeology
☐	☒	Animals and other organisms
☐	☒	Human research participants
☒	☐	Clinical data
☒	☐	Dual use research of concern

Methods

n/a	Involved in the study	
☒	☐	ChIP-seq
☒	☐	Flow cytometry
☒	☐	MRI-based neuroimaging

Antibodies

Antibodies used

anti-GIV-coiled-coil: Rabbit polyclonal, EMD Millipore (Cat# ABT80), 1:500 (WB), anti-AMPKalpha2: Rabbit polyclonal, Abcam (Cat# 3760, lot# GR141300-27), 1:500 (WB), phospho-AMPKalpha (Thr172) (40H9): Rabbit monoclonal, Cell Signaling Technology (Cat# 2535, lot# 16), 1:1000 (WB), anti-AMPKbeta1: Rabbit polyclonal, Abcam (Cat# 217348), 1:50 (IHC), anti-Claudin-2: Rabbit polyclonal, Abcam (Cat# 53032, lot# GR314368-12), 1:100 (IHC-mouse), 1:250 (IHC-human), anti-Occludin (OC-3F10): Mouse monoclonal, Invitrogen (Cat# 33-1500, lot# TJ275405), 1:300 (IF), phospho-S245 GIV, Rabbit polyclonal, 21st Century Biochemicals (Custom antibody), 1:500 (WB), 1:300 (IF), 1:50 (IHC), anti-alphaTubulin (DM1A): Mouse monoclonal, Sigma (Cat# 9026), 1:500 (WB) anti-myeloperoxidase: Rabbit polyclonal, Abcam (Cat# 9535), 1:30 (IHC), Goat anti-rabbit alexa fluor 594, Invitrogene (Cat#A32740), Rabbit polyclonol), 1:500 (IF), Goat anti-rabbit IRDye 680, LI-COR, (Cat#926, 68071) 1:10000 (WB), Goat anti-mouse IRDye 800, LI-COR, (Cat#926, 32210) 1:10000 (WB).

Validation

anti-GIV-coiled-coil: Rabbit polyclonal, EMD Millipore (Cat# ABT80): This antibody has been validated by the supplier for immunoblot and has reactivity against mouse and human. https://www.emdmillipore.com/US/en/product/Anti-Girdin-coiled-coil-region-Antibody,MM_NF-ABT80
anti-AMPKalpha2: Rabbit polyclonal, Abcam (Cat# 3760, lot# GR141300-27): This antibody has been validated by the supplier for immunoblot and has reactivity against mouse and human. https://www.abcam.com/ampk-alpha-2-antibody-ab3760.html. Antibody validated by immunoblot using WT and AMPK KO enteroid derived monolayers (EDMs) in Supplementary Figure S13 of this manuscript.
phospho-AMPKalpha (Thr172) (40H9): Rabbit monoclonal, Cell Signaling Technology (Cat# 2535, lot# 16): This antibody has been validated by the supplier for immunoblot and has reactivity against mouse and human. https://www.cellsignal.com/products/primary-antibodies/phospho-ampka-thr172-40h9-rabbit-mab/2535. Antibody validated by immunoblot using WT and AMPK KO enteroid derived monolayers (EDMs) in Supplementary Figure S13 of this manuscript.
anti-AMPKbeta1: Rabbit polyclonal, Abcam (Cat# 217348): This antibody was validated by the supplier for IHC and has reactivity against mouse and human. https://www.abcam.com/ampk-beta-1-antibody-ab217348.html.
anti-Claudin-2: Rabbit polyclonal, Abcam (Cat# 53032, lot# GR314368-12): This antibody was validated by the supplier for IHC and has reactivity against mouse and human. https://www.abcam.com/claudin-2-antibody-ab53032.html.
anti-Occludin (OC-3F10): Mouse monoclonal, Invitrogen (Cat# 33-1500, lot# TJ275405): This antibody was validated by the supplier for immunofluorescence and has reactivity against mouse and human. https://www.thermofisher.com/antibody/product/Occludin-Antibody-clone-OC-3F10-Monoclonal/33-1500.
Phospho-S245 GIV, Rabbit polyclonal, 21st Century Biochemicals (Custom antibody): This antibody was validated by our group for immunoblot and immunofluorescence in previously published work (Aznar et. al., eLife 2016, https://elifesciences.org/articles/20795) and has reactivity against mouse.
anti-alphaTubulin (DM1A): Mouse monoclonal, Sigma: This antibody has been validated by the supplier for immunoblot and has reactivity against mouse and human. https://www.sigmaaldrich.com/catalog/product/sigma/t9026?lang=en&region=US.

anti-myeloperoxidase: Rabbit polyclonal, Abcam: This antibody was validated by the supplier for IHC and has reactivity against mouse and human. https://www.abcam.com/myeloperoxidase-antibody-ab9535.html.

Eukaryotic cell lines

Policy information about cell lines

Cell line source(s)	*State the source of each cell line used.*
Authentication	*Describe the authentication procedures for each cell line used OR declare that none of the cell lines used were authenticated.*
Mycoplasma contamination	*Confirm that all cell lines tested negative for mycoplasma contamination OR describe the results of the testing for mycoplasma contamination OR declare that the cell lines were not tested for mycoplasma contamination.*
Commonly misidentified lines (See ICLAC register)	*Name any commonly misidentified cell lines used in the study and provide a rationale for their use.*

Animals and other organisms

Policy information about studies involving animals; ARRIVE guidelines recommended for reporting animal research

Laboratory animals	Intestinal crypts were isolated either from the proximal and the mid-colon of WT C57BL/6 or AMPK KO mice; generated from gender- and age-matched littermates of age 5-7 weeks. For DSS-colitis experiments, 7-8-wk old C57Bl/6 mice were obtained from Jackson Laboratories (Bay Harbor, ME). Animals were bred, housed (light and dark cycle of 12 h each, humidity 30-70% and room temperature controlled between 68-75 °F), and euthanized according to University of California San Diego Institutional Animal Care and Use Committee (IACUC) policies and guidelines.
Wild animals	This study did not involve wild animals.
Field-collected samples	This study did not involve samples collected from the field.
Ethics oversight	Animals were bred, housed, and euthanized according to University of California San Diego Institutional Animal Care and Use Committee (IACUC) policies and guidelines.

Note that full information on the approval of the study protocol must also be provided in the manuscript.

Human research participants

Policy information about studies involving human research participants

Population characteristics

Healthy controls:
Age: 0-64 years
Gender: both male and female.
Ethnicity: Caucasian, Hispanic
Disease Location: Not applicable.
Disease Duration: Not applicable.
Drug history: Not applicable.

Ulcerative colitis patients :
Age: 19-47 years.
Gender: both male and female.
Ethnicity: Caucasian and Asian.
Disease location: Pancolitis and Left sided colitis.
Disease Duration: 0.28-21 years.
Drug history: case dependent, using of Adalimumab, ASA, Infliximab, and Vedolizumab.

Chron's disease patients:
Age : 27-71 years.
Gender: both male and female.
Ethnicity: Caucasian, African American and Middle Eastern.
Disease location: Ileocolitis, colitis, and Ileitis including stricturing, non-stricturing, non-penetrating, and penetrating.
Disease Duration: 1-23 years.
Drug history: case dependent, either naive or include the use of Remicade, Humira, Adalimumab, Infliximab, and Vedolizumab. Some cases have past history of Adalimumab, Infliximab, and Vedolizumab.

Recruitment	For immunohistochemical analysis of human tissue specimens, archived formaldehyde-fixed paraffin-embedded (FFPE) human colonic biopsies from healthy controls were obtained from the Gastroenterology Division, VA San Diego Healthcare System, following the protocol approved by the Human Research Protection Program (HRPP) Institutional Review Board (Project ID# 1132632).<br>Colonic biopsies used as a source of stem cells for organoid culture were obtained from healthy and IBD patients undergoing colonoscopies a part of their routine care and follow-up at UC San Diego's Inflammatory Bowel Disease (IBD) Center. Patients were recruited and consented using a study proposal approved by the Institutional Review Board of the University of California, San Diego. The clinical phenotype and information were curated based on histopathology reports from Clinical Pathology and Chart check, followed by consultation with a specialist at UC San Diego's IBD Center. The specimens were collected following the protocol approved by the Human Research Protection Program (HRPP) Institutional Review Board (Project ID#131487 for UCSD Inflammatory Bowel Disease Center Patient Biobank and Project ID#190105 for the HUMANOID CoRE).<br>For the recruitment, subjects are identified by the treating physician who has a deep understanding of the pathophysiology and clinical practice guidelines when it comes to the GI conditions. The process of identification will involve checking the patient's chart for study eligibility, mainly why the procedure was scheduled by the patient's physician (i.e., indication for procedure as stated in chart) to judge if the patient meet's inclusion criteria, and whether existing co-morbidities warrant exclusion from the study. We have used the following inclusion and exclusion criteria.<br>Inclusion Criteria: Consented to the study; Healthy or diseased, where tissue can be endoscopically accessed; Patients presenting with routine requests for diagnostic or screening endoscopic procedures; any gender, ethnicity and socio-economic background.<br>Exclusion Criteria: Impaired decisional capacity, comprehension, cognition; Known risk of bleeding (with the exception of patients on low-dose aspirin), in which case, any additional biopsies/samples may increase the risk of complications, e.g., patient on blood thinners, known bleeding diathesis, such as von Willebrand's disease or hemophilla; Any other illness that impairs, as a secondary complication platelet function or number that may increase risk of bleeding due to additional biopsies; when taking samples for research will impact the ability to perform accurate histopathologic diagnosis for clinical care (i.e., extremely small lesions, where excess tissue is not available); Pregnancy (beta HCG testing is done routinely before endoscopy); Declined consent.<br>Patients are consented at least 2 hours prior to the procedure either in the pre-procedure room or in the procedure room. Moreover, study subjects are enrolled at the time when they present for routine endoscopic procedures (interrogating the upper as well as lower GI tract) after taking the necessary preparation for such a procedure. They have to have undergone prior assessment for fitness to undergo the procedure as per routine procedure guidelines. The procedures should have been ordered during the course of routine standard of care, as determined by their treating physicians, with no influence whatsoever from the endoscopist who will enroll patients into the study and obtain informed consent. In many cases, several healthy individuals undergoing routine screening procedures are consented as potential sources for healthy "control/normal" tissues but may be found to have disease conditions. Thus, informed consent includes such disclosure that depending on the endoscopic findings, the nature of participation in the study may change in those cases. The risk is low because the decision to get the procedure done and the indication is determined by another physician/team other than the PI who is conducting this study/obtaining informed consent. However, to further minimize risk, it is clarified that the decision to participate in the study is entirely voluntary and can not affect management/care decisions. i.e. the study participant can not get any different care if they choose not to participate.
Ethics oversight	University of California San Diego, Human Research Protection Program (HRPP) Institutional Review Board Project no 190105, 1132632, 131487.

Note that full information on the approval of the study protocol must also be provided in the manuscript.
