# Supplementary figures and images for: Artificial intelligence guided discovery of a barrier-protective therapy in inflammatory bowel disease
Source: Nat Commun. 2021 Jul 12;12:4246. doi: 10.1038/s41467-021-24470-5 (PMC8275683; doi:10.1038/s41467-021-24470-5)

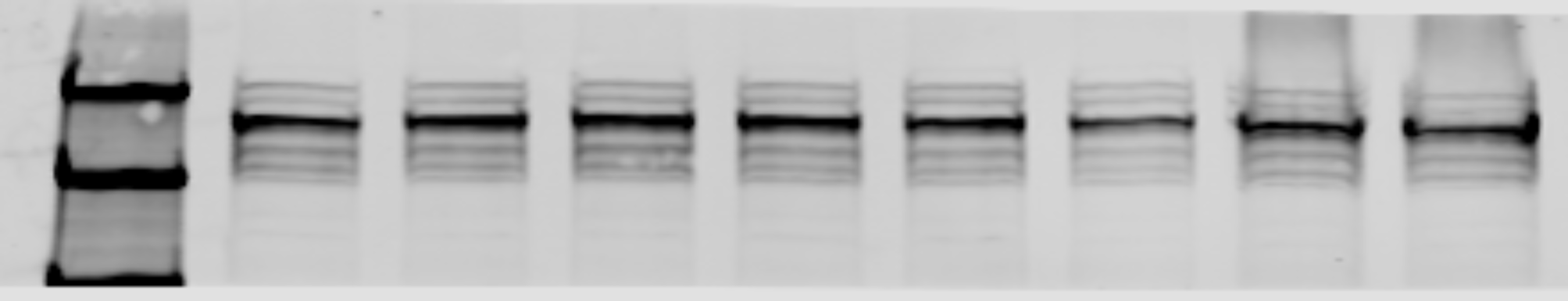

Supplement: Supplementary file 9 — Source Data [file 41467_2021_24470_MOESM9_ESM.zip › Source Data/Fig S13 B_EDM whole blots copy/EDM_un_met_A7_PF_GIVcc.tif]

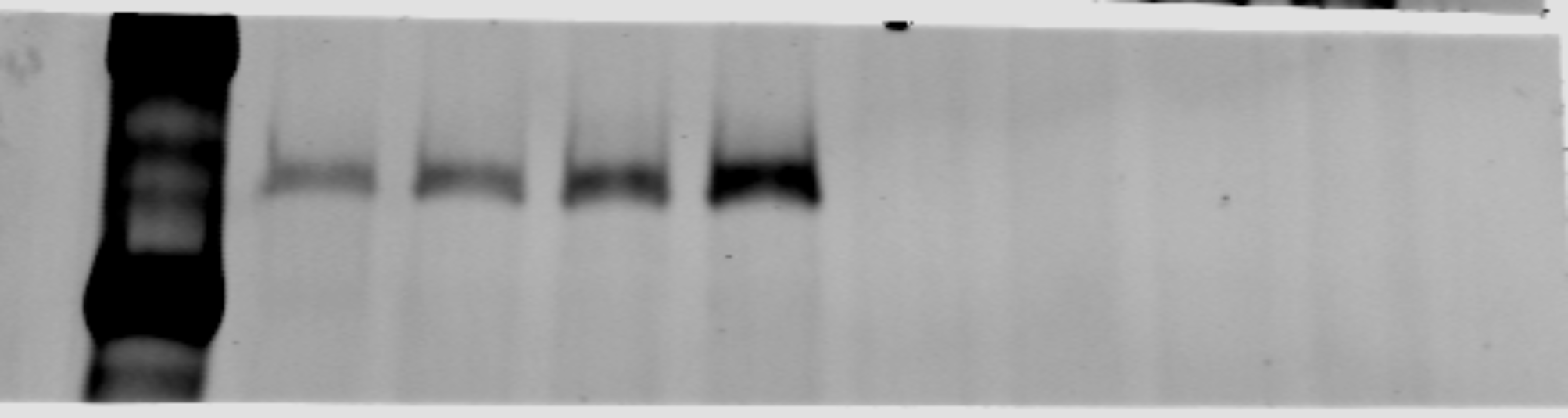

Supplement: Supplementary file 9 — Source Data [file 41467_2021_24470_MOESM9_ESM.zip › Source Data/Fig S13 B_EDM whole blots copy/EDM_un_met_A7_PF_phosAMPK.tif]

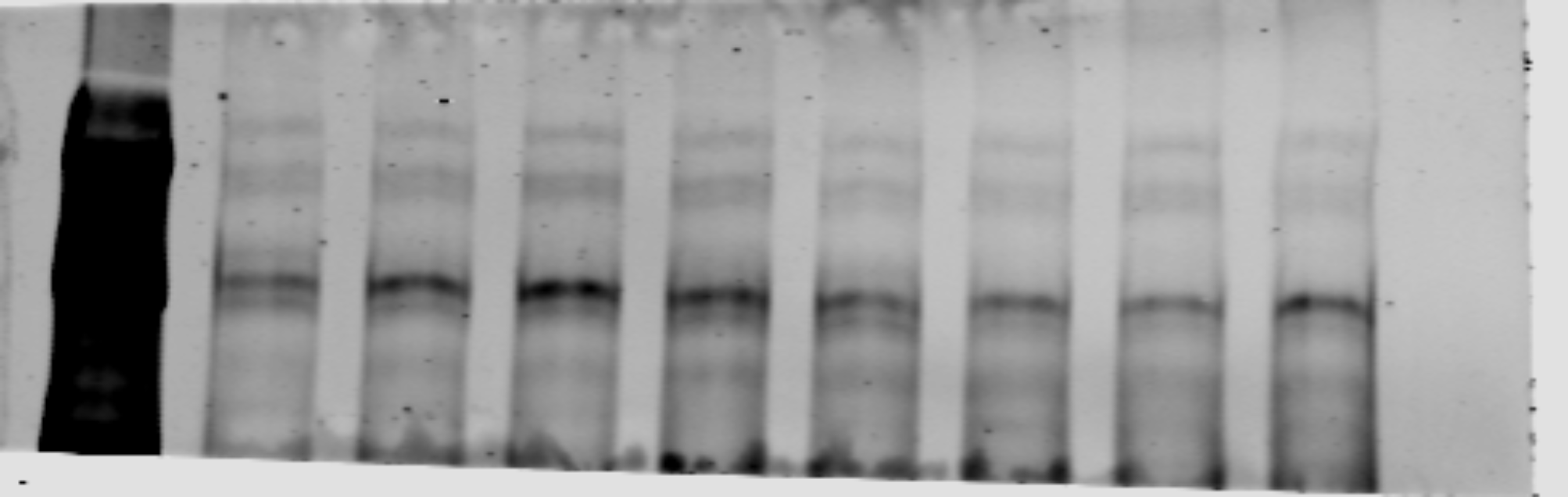

Supplement: Supplementary file 9 — Source Data [file 41467_2021_24470_MOESM9_ESM.zip › Source Data/Fig S13 B_EDM whole blots copy/EDM_un_met_A7_PF_pS245.tif]

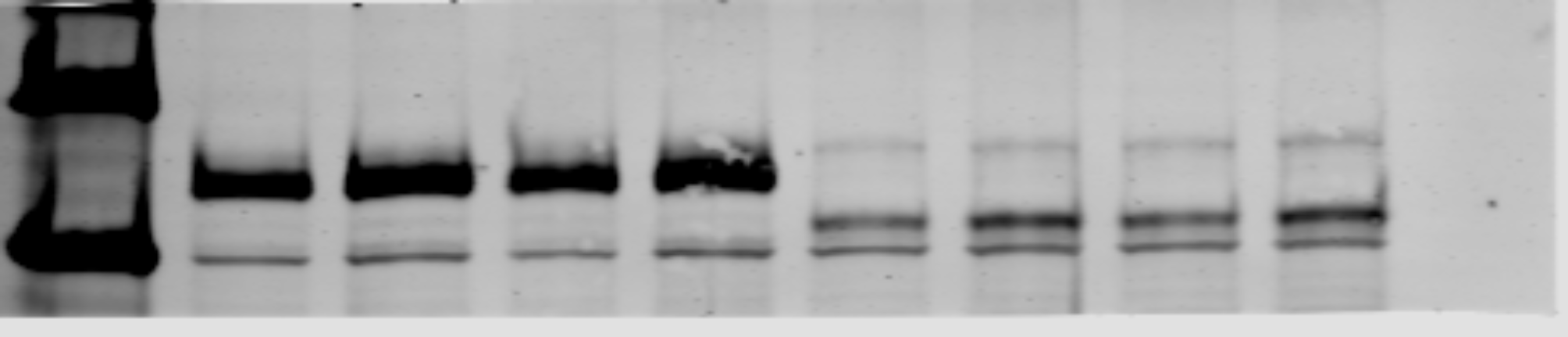

Supplement: Supplementary file 9 — Source Data [file 41467_2021_24470_MOESM9_ESM.zip › Source Data/Fig S13 B_EDM whole blots copy/EDM_un_met_A7_PF_tAMPK.tif]

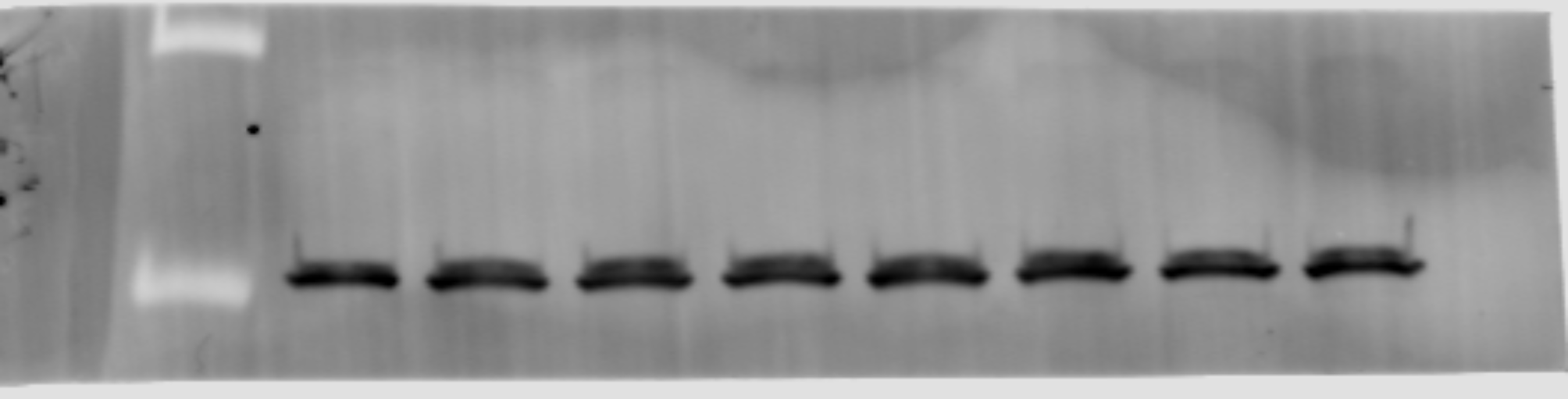

Supplement: Supplementary file 9 — Source Data [file 41467_2021_24470_MOESM9_ESM.zip › Source Data/Fig S13 B_EDM whole blots copy/EDM_un_met_A7_PF_tubulin.tif]

## Slide 1
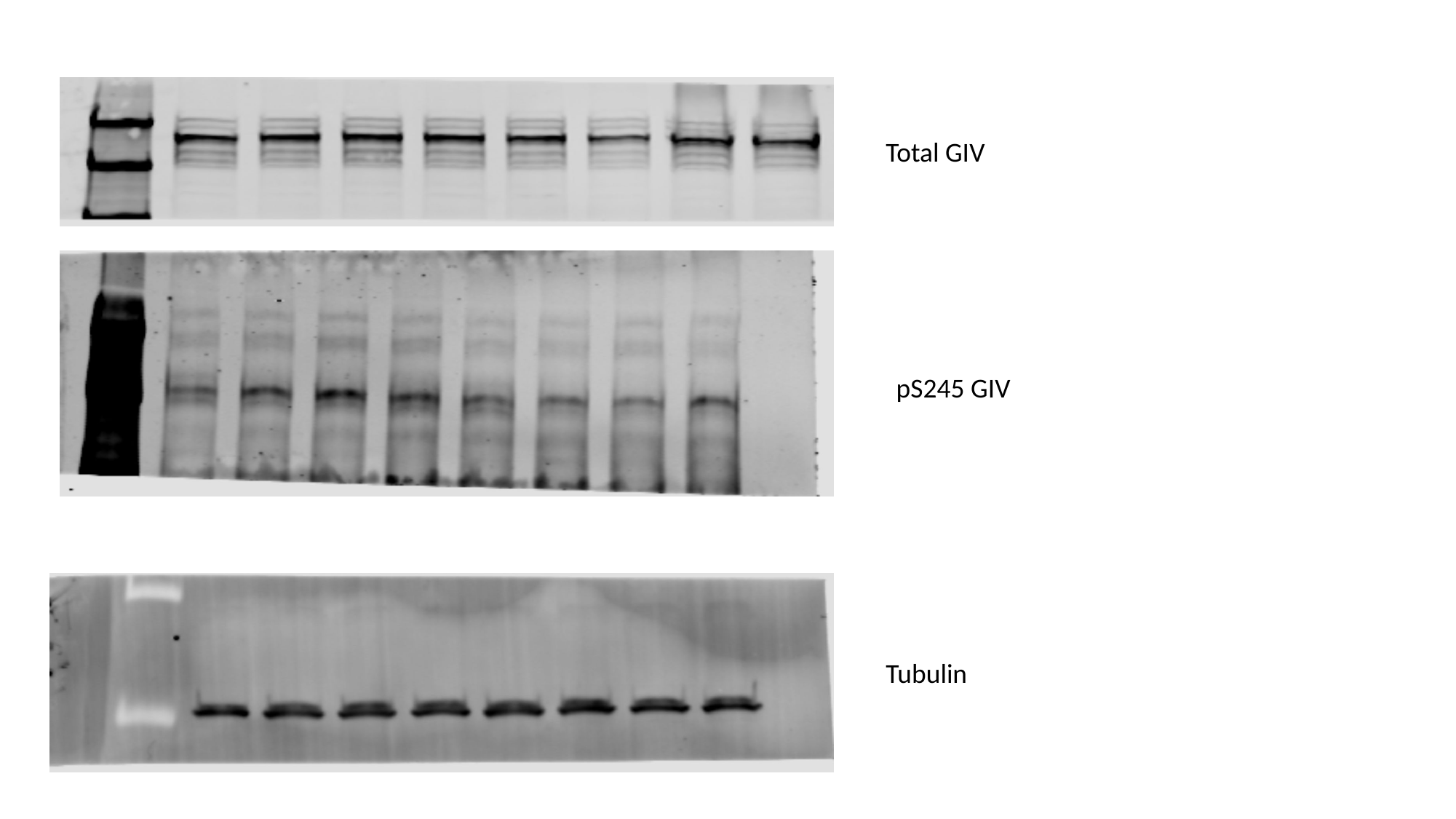

Total GIV
pS245 GIV
Tubulin

## Slide 2
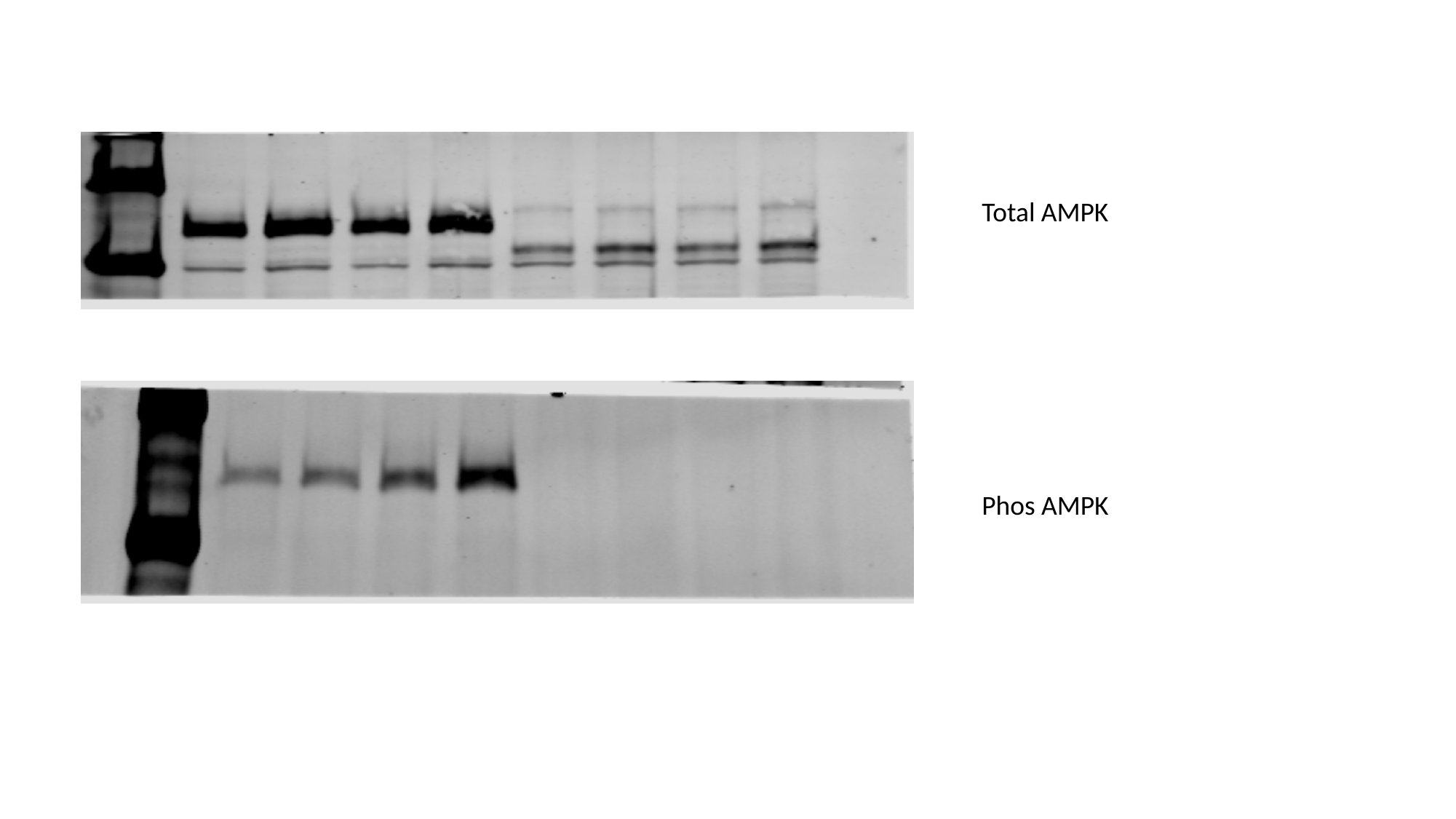

Total AMPK
Phos AMPK

Supplement: Supplementary file 9 — Source Data [file 41467_2021_24470_MOESM9_ESM.zip › Source Data/Fig S13 B_EDM whole blots copy/EDM_un_met_A7_PF_whole blots.pptx]
